# VICTOR: genome-based phylogeny and classification of prokaryotic viruses

**DOI:** 10.1093/bioinformatics/btx440

**Published:** 2017-07-07

**Authors:** Jan P Meier-Kolthoff, Markus Göker

**Affiliations:** Department of Bioinformatics, Leibniz Institute DSMZ – German Collection of Microorganisms and Cell Cultures, Braunschweig, Germany

## Abstract

**Motivation:**

Bacterial and archaeal viruses are crucial for global biogeochemical cycles and might well be game-changing therapeutic agents in the fight against multi-resistant pathogens. Nevertheless, it is still unclear how to best use genome sequence data for a fast, universal and accurate taxonomic classification of such viruses.

**Results:**

We here present a novel *in silico* framework for phylogeny and classification of prokaryotic viruses, in line with the principles of phylogenetic systematics, and using a large reference dataset of officially classified viruses. The resulting trees revealed a high agreement with the classification. Except for low resolution at the family level, the majority of taxa was well supported as monophyletic. Clusters obtained with distance thresholds chosen for maximizing taxonomic agreement appeared phylogenetically reasonable, too. Analysis of an expanded dataset, containing >4000 genomes from public databases, revealed a large number of novel species, genera, subfamilies and families.

**Availability and implementation:**

The selected methods are available as the easy-to-use web service ‘VICTOR’ at https://victor.dsmz.de.

**Supplementary information:**

Supplementary data are available at *Bioinformatics* online.

## 1 Introduction

Viruses are ancient structures (Forterre, 2006), infect organisms of all three domains of life and outnumber the cells of their hosts by one to two orders of magnitude (Suttle, 2007). The impact of viruses on the global biogeochemical cycles and thus on life on earth is immense (Suttle, 2007). Since prokaryotes are the most abundant organisms on this planet (Whitman *et al.*, 1998), they are the most common hosts of viruses. Consequently, bacterial and archaeal viruses are the most abundant biological entities, not only in the virosphere itself but on earth in general (Koonin *et al.*, 2015). Even though the genetic diversity of these viruses is unmatched (Hendrix, 2003; Kristensen *et al.*, 2013; Prangishvili *et al.*, 2006), probably only a minor fraction of them are known (Rohwer, 2003). In recent years, the interest in bacterial viruses (bacteriophages) as therapeutic agents to fight serious infections caused by multi-resistant strains (Abedon *et al.*, 2011) experienced a renaissance (Thacker, 2003). Likewise, the role of viruses in aquatic ecosystems (Wommack and Colwell, 2000), especially by infecting and lysing aquatic microorganisms, and their subsequent impact on life-cycle and evolution of abundant marine groups such as *Rhodobacteraceae* (Simon *et al.*, 2017), is still not fully understood.

A reliable classification of the ever increasing number of known prokaryotic viruses (Ackermann, 2007) is thus of utmost importance. The International Committee on Taxonomy of Viruses (ICTV) (Adams *et al.*, 2017) established rules for the naming and classification of viruses, represented by the International Code of Virus Classification and Nomenclature (Adams *et al.*, 2017). Viruses are taxonomically arranged by a variety of characteristics (Ackermann, 2009), and the affiliation of prokaryotic viruses to the same family is currently possible despite a complete lack of DNA sequence relatedness (Nelson, 2004). A stronger role of genome sequences in virus classification was recommended though (Krupovic *et al.*, 2016; Simmonds *et al.*, 2017). As of March 2015, the ICTV master species list recognizes prokaryotic viruses assigned to 548 species, 103 genera, 7 subfamilies and 18 families but a much larger number of genomes of bacterial and archaeal viruses is available in public databases.

DNA:DNA hybridization experiments with phages were already conducted by Grimont and Grimont (1981), who found stable hybridization groups and proposed to define a phage species as a group of phages with ‘significant’ genomic relatedness. This technique is not routinely applied anymore due to an emphasis on genomics (Ackermann, 2009), and, in contrast to the classification of *Bacteria* and *Archaea*, virus taxonomy has not yet established firm distance or similarity thresholds for assigning viral genomes to a taxon of a given taxonomic rank (Krupovic *et al.*, 2016). In microbiology, the 70% DNA:DNA hybridization threshold has been applied since decades as the ultimate criterion to separate prokaryotic species (Brenner, 1973; De Ley, 1970; Johnson, 1973; Wayne *et al.*, 1987). It was successfully replaced recently by bioinformatic techniques applied to partial or complete genome sequences (Auch *et al.*, 2010a; Meier-Kolthoff *et al.*, 2013). Boundaries for the subspecies rank have also been suggested (Meier-Kolthoff *et al.*, 2014a), based on the same kind of intergenomic distances calculated with the Genome BLAST Distance Phylogeny (GBDP) tool (Auch *et al.*, 2010a,b; Henz *et al.*, 2005). These techniques were made available as an easy-to-use web service (Auch *et al.*, 2010a,b).

Several sequence-based approaches were specifically designed to tackle phage classification (Rohwer and Edwards, 2002) but sometimes addressing the genus and family level only (Lima-Mendez *et al.*, 2008; Roux *et al.*, 2015) or restricted to a single taxonomic rank within a particular virus family (Lavigne *et al.*, 2008) or subfamily (Asare *et al.*, 2015). Bacteriophages can be classified according to the inferred organization of their neck region (Lopes *et al.*, 2014) but this is applicable to tailed phages only. Some tools (Asare *et al.*, 2015; Ågren *et al.*, 2012; Frazer *et al.*, 2004) were recently suggested by Krupovic *et al.* (2016) for virus taxonomy but these were not primarily devised for this purpose and are thus not necessarily optimal. Clustering techniques were frequently applied but not necessarily optimized for virus classification, with the notable exceptions of Lima-Mendez *et al.* (2008) and Roux *et al.* (2015). Only few methods attempted to incorporate phylogenetic analyses but these either were based on a predefined selection of few genes (Asare *et al.*, 2015) or inferred trees but no branch support (Rohwer and Edwards, 2002). Methods using standard sequence-alignment techniques (Ceyssens *et al.*, 2011; Lavigne *et al.*, 2008) only work with collinear viral genomes. Most of the suggested approaches were restricted to amino-acid sequences (Ågren *et al.*, 2012; Lavigne *et al.*, 2008; Lima-Mendez *et al.*, 2008; Lopes *et al.*, 2014; Roux *et al.*, 2015), and methods based on gene content were seldom compared with those based on sequence similarity (Rohwer and Edwards, 2002).

Thus a comprehensive optimization of methods for the classification of prokaryotic viruses, covering both clustering techniques and phylogenetic inference (including branch support), nucleotide and amino-acid sequences, and gene-content based, sequence-similarity based and combined strategies, appears to be missing. GBDP is promising in this respect because of its variety of options for determining homologous regions between genomes (Auch *et al.*, 2010a; Meier-Kolthoff *et al.*, 2013, 2014b), its algorithms for correcting for paralogy (Henz *et al.*, 2005), the variety of GBDP distance formulas that explore distinct genomic features (Auch *et al.*, 2010a; Meier-Kolthoff *et al.*, 2013, 2014b), and the ability to infer phylogenetic trees including branch-support values based on resampling (Meier-Kolthoff *et al.*, 2014b).

Indeed, clustering based on sequence or distance thresholds on the one hand and phylogenetic inference on the other hand may be in conflict with each other. When distance matrices deviate from ultrametricity (Swofford *et al.*, 1996), which usually happens if the evolution of the underlying sequence data deviated from a molecular clock (Felsenstein, 2004), clusters based on a given distance or similarity threshold might not correspond to monophyletic groups (Wood, 1994). The principles of phylogenetic systematics imply that the goal of taxonomic classification is to summarize the phylogeny of the organisms under study (Hennig, 1965; Wiley and Lieberman, 2011). Non-monophyletic taxa (Farris, 1974) are in conflict with that goal (Hennig, 1965; Wiley and Lieberman, 2011); the ideal classification comprises only taxa that are statistically well supported as monophyletic (Vences *et al.*, 2013). Whereas ultimately rather a problem of the data and not of the methods, the severity of taxonomic problems due to non-ultrametricity depends on the organisms under study, the characters collected from these organisms and the methods used to analyse those characters. For instance, when using intergenomic distances inferred with GBDP to assign *Archaea* and *Bacteria* to species, few issues caused by non-ultrametric data were found (Meier-Kolthoff *et al.*, 2014c). The same holds for subspecies, provided the distance or similarity threshold (and clustering approach) is chosen for maximum cluster consistency (Meier-Kolthoff *et al.*, 2014a).

To create an easy-to-use web service for phylogeny and classification of prokaryotic viruses, in this study we optimize the combination of GBDP parameters, analysed at both nucleotide and amino-acid level, to yield as many virus taxa highly supported as monophyletic as possible, quantified as ‘taxon support’. The ICTV classification, combined with accordingly taxonomically annotated viral genome sequences from public repositories, is used as reference dataset. As secondary criterion, we optimize GBDP settings, clustering algorithms and distance thresholds to yield the highest agreement with the ICTV assignment of the virus genomes to taxa at distinct ranks, quantified via the Modified Rand Index (MRI) as implemented in OPTSIL (Göker *et al.*, 2009). The results are discussed regarding the proportion of taxa that could be kept as-is, the number of revisions needed when broadly implementing the standardized approach and other potentials and limitations of the suggested method, such as those ranks too high to be resolved any more using GBDP. As an example for applying the devised approach to phylogeny and classification, we analyse an expanded dataset of virtually all publicly available genomes of prokaryotic viruses. The outcome is quantified regarding the number of overall, known and novel taxa and regarding the detectable host specificity. Our phylogenomic approach contributes to understanding the composition of the viral biosphere by means of an accurate phylogenetic inference and classification.

## 2 Materials and methods

### 2.1 Reference dataset

Genomes were taxonomically selected by querying the INSDC databases for all species names assigned to families of prokaryotic virus in the third version of the 2014 ICTV master species list (https://talk.ictvonline.org/files/master-species-lists/) (King *et al.*, 2012), which contained a total of 548 species, 103 genera, 7 subfamilies and 18 families; we did not observe a new version in 2017 that contained more taxa. Using all available whole-genome sequences of prokaryotic viruses instead would enrich the dataset with informal taxon names that could hardly be compared with each other and to the formally accepted names in the ICTV master list. Genomes assigned to species *sensu lato* were also removed. The collected data were further restricted to complete genome sequences containing protein annotation. Duplicate genomes (due to distinct annotation versions) were detected using MD5 checksums calculated from their nucleotide sequences and only the version with most protein sequences kept. The reference dataset is listed in Supplementary File S1.

### 2.2 Distance calculation

Pairwise intergenomic distances were calculated between amino acid and nucleotide sequences with the current version of the GBDP approach (Meier-Kolthoff *et al.*, 2013), including 100 pseudo-bootstrap replicates (Meier-Kolthoff *et al.*, 2014b) for calculating branch support. BLAST+ (Camacho *et al.*, 2009) was used as local alignment tool under default settings but with a broad range of e-value filters (10, 1, 1e-1, 1e-2, 1e-3, 1e-8). GBDP was run with two distinct algorithms (trimming and coverage) for filtering the BLAST+ output as well as its ten distance formulas (Meier-Kolthoff *et al.*, 2013) for exploring either gene content or sequence identity or both. Thus a total of 240 unique combinations of parameters were investigated (2 types of sequence data × 6 e-value settings × 2 GBDP algorithms × 10 GBDP distance formulas). All settings are included in Supplementary File S1 together with the respective results.

### 2.3 Inference and assessment of phylogenetic trees

Phylogenetic trees were inferred from the original and pseudo-bootstrapped distance matrices using SPR branch swapping as implemented in FastME 2.1.4 (Lefort *et al.*, 2015) and rooted using the midpoint method (Hess and De Moraes Russo, 2007). FastME is topologically more accurate than the well-known neighbour-joining (NJ) algorithm (Saitou and Nei, 1987) because both are based on the balanced minimum evolution criterion, which NJ only greedily optimizes (Lefort *et al.*, 2015). Checks for monophyletic, paraphyletic and polyphyletic taxa were conducted using the criteria of Farris (1974) and in-house developed scripts (Hahnke *et al.*, 2016). The bootstrap support of each non-trivially monophyletic taxon was recorded, as well as the support against each non-monophyletic taxon, quantified as the maximum support among all clades in the rooted phylogeny that were in conflict with the monophyly of that taxon. The sum of the support values for the taxa at each taxonomic rank, relative to the sum of all support values, either for or against the monophyly of a taxon, yielded a measure of overall phylogenetic fit (‘taxon support’) of a given tree, and thus of the underlying distance matrix and combination of GBDP parameters, to the taxonomic classification at that rank. If most taxa of a certain taxonomic category in the ICTV classification (King *et al.*, 2012) received little positive or negative support, this would indicate insufficient resolution of the respective method at that rank, whereas strong support against many taxa would indicate a bias of the method. In contrast, single taxa with strong support against their monophyly rather pointed to a problem with the classification or just with the naming of certain INSDC entries.

### 2.4 Inference and assessment of clusterings

In clustering experiments additional to phylogenetic inference, the OPTSIL software (Göker *et al.*, 2009) was used to optimize distance thresholds *T* by maximizing the agreement of the resulting non-hierarchical clustering with a given reference partition. Agreement was quantified as the MRI (Göker *et al.*, 2009), which is equal to 1 in the case of two identical partitions (Hubert and Arabie, 1985) and proportionally lower depending on the amount of disagreement. The ICTV classification reduced to each rank was used as reference partition; genomes from species not assigned to a genus were removed when analysing the genus rank. The *F* parameter of OPTSIL was set to 0.5, which yielded the highest clustering consistency in earlier studies (Meier-Kolthoff *et al.*, 2014a). The fit of each distance matrix, and thus each combination of GBDP parameters, to the taxonomic classification at each rank can then be represented as the highest obtained MRI value. However, clusters inferred from the same distance matrix as a phylogenetic tree that yielded strong support against their monophyly would point to problems due to non-ultrametricity in the data (Meier-Kolthoff *et al.*, 2014c). For this reason, taxon support for the optimal clusters was determined as described above for the ICTV taxa.

### 2.5 Correspondence between criteria and optimal settings

Since each of the three taxonomic ranks yielded a separate optimality criterion regarding both taxon support and MRI, the correspondence between the resulting six optimality criteria was explored with a principal-components analysis as implemented in the FactoMineR package (Lê *et al.*, 2008) for the statistical environment R Development Core Team (2015). A Pareto multi-objective selection was conducted with the rPref package (Roocks, 2016) for R Development Core Team (2015) to determine the subset of equally feasible choices (‘Pareto frontier’) for nucleotide and amino-acid sequences, respectively. The taxon support for each of the three ranks family, genus and species served as the first set of objectives; the resulting subset of GBDP settings was further reduced using the MRI values as second set of objectives. R code to analyse and plot the optimality criteria is provided in Supplementary File S2.

### 2.6 Delineation of taxa at the subfamily level

The subfamily category is currently only incompletely applied in virus taxonomy; a subfamily had been assigned to only 87 viruses within our reference dataset. Thus subfamilies were not considered when optimizing GBDP settings. Instead, the performance of the optimal settings was assessed separately regarding subfamilies using a dataset reduced to the 87 genomes, and the best subfamily specific clustering thresholds determined only for these previously chosen GBDP settings.

### 2.7 Analysis of expanded dataset

A larger dataset of 4419 prokaryotic viruses was collected from GenBank and the PhAnToMe FTP server (as of July 2016) (Overbeek *et al.*, 2014), without restriction to taxa recognized by the ICTV (Supplementary File S1). Using the best GBDP settings for the analysis of amino-acid sequences and the thresholds for delineation at the species, genus, subfamily and family rank, virus diversity was quantified and compared with the diversity found in the reference dataset. In addition to the number of new and already known clusters or taxa and the number of genomes per cluster or taxon, the effect of increased genome sampling on host specificity was examined. The ‘specific host’ entry was extracted from the GenBank files and restricted to validly published names of host taxa as listed in Prokaryotic Nomenclature Up-To-Date (October 2016, https://www.dsmz.de/bacterial-diversity/prokaryotic-nomenclature-up-to-date.html). The thus standardized host names were used as-is; fixing prokaryotic taxa that do not reflect their phylogenetic relationships (Klenk and Göker, 2010) was beyond the scope of the present study. Finally, the host specificity of each cluster was assessed in analogy to the Berger-Parker-Index (Berger and Parker, 1970) via the formula *m*/*N* with *m* being the frequency of the most frequent host and *N* the total number of (potential) hosts indicated for the genomes in that cluster. The dependency of this index on *N* was studied using robust line fits as implemented in R Development Core Team (2015) since high specificity might be a sampling artefact.

## 3 Results

### 3.1 Optimal GBDP settings

The reference dataset included 610 genomes (Supplementary File S1). The total number of pairwise intergenomic distances was c. 4.5B (185 745 pairwise comparisons × 100 replicates × 240 parameter combinations). The average branch support ranged from 25 to 97% (median: 50%) for the 120 amino-acid trees and from 2 to 92% (median: 42%) for the 120 nucleotide trees. For applying OPTSIL to the genus rank, the set had to be reduced to 588 genomes (i.e. 22 viruses had no genus affiliation).

**Fig. 1.**
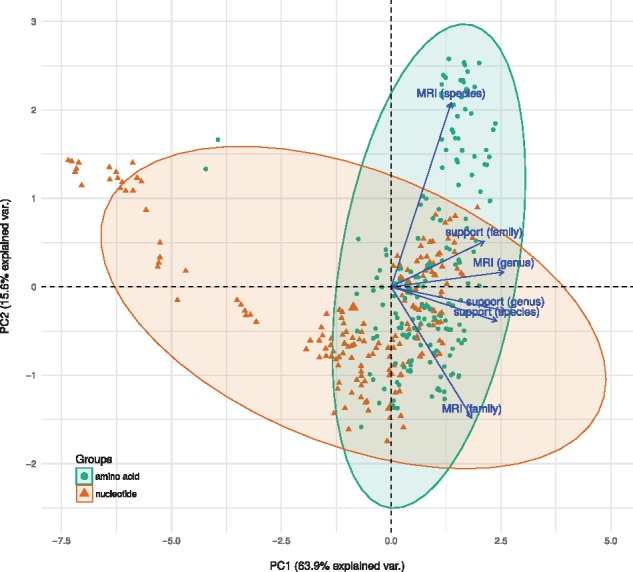
Relative performance of GBDP settings and optimality criteria. The two principal components (PC1 and PC2) that explained most of the variation (given in percent) in the data are displayed. Dots represent the individual GBDP settings, whereas arrows represent the loadings of the relevant variables and thus the performance of each combination of GBDP parameters. Normal probability ellipsoids for 95% confidence limits are shown separately for amino acid and nucleotide sequences (Color version of this figure is available at *Bioinformatics* online.)

The biplot of the two most important principal components in Figure 1 shows the relative directions and loadings of the six optimality criteria (Supplementary File S1). Apparently, the majority of the criteria, including all taxon-support objectives, indicate approximately the same optimal GBDP settings, whereas MRI values for species and family, respectively, point into distinct directions. Table 1 shows the GBDP settings that yielded the highest taxon support and, among those, the GBDP settings and according distance threshold (*T*) values that yielded the highest MRI values. Nucleotide and amino-acid sequences both favored the greedy-with-trimming algorithm (Meier-Kolthoff *et al.*, 2014b) but required distinct distance formulas, BLAST+ (Camacho *et al.*, 2009) word lengths and e-values; amino-acid sequences yielded (i) higher average branch support, (ii) marginally lower taxon support values for species and genera, (iii) a pronounced lower taxon support value for families, (iv) similarly high MRI values for species and genera and (v) a higher (but overall still comparatively low) MRI value for the family rank.

**Table 1.** Optimal GBDP settings

	Amino acid	Nucleotide
Word length	3	11
E-value filter	0.1	1.0
Algorithm	Greedy-with- trimming	Greedy-with-trimming
Formula	D6	D0
Average support	56.52%	36.77%
Number of species	480	480 (same as left col.)
Number of genera	98	98 (same as left col.)
Number of families	15	15 (same as left col.)
Taxon support, species	0.87	0.90
Taxon support, genus	0.95	0.96
Taxon support, family	0.47	0.73
MRI, species	0.67	0.67
MRI, genus	0.89	0.89
MRI, family	0.72	0.34
*T*, species	0.118980	0.022085
*T*, genus	0.749680	0.842700
*T*, family	0.985225	0.997455
Number of species clusters	387	474
Number of genus clusters	115	132
Number of family clusters	10	32
Taxon support, species clusters	0.99	0.98
Taxon support, genus clusters	1.00	0.99
Taxon support, family clusters	0.42	0.82
Species to be split	2.30%	2.50%
Genera to be split	9.10%	16.20%
Families to be split	33.30%	40.00%
Multi-species cluster	17.10%	4.60%
Multi-genus clusters	7.80%	4.50%
Multi-family clusters	70.00%	37.50%

*Note*: The best GBDP settings according to the two-step Pareto multi-objective optimization based on (i) taxon support and (ii) MRI at each taxonomic rank, after optimizing the distance threshold *T* separately for each rank and an *F* value of 0.5. Numeric results for the ICTV reference datasets are also provided.

### 3.2 Remaining non-monophyletic taxa

**Fig. 2.**
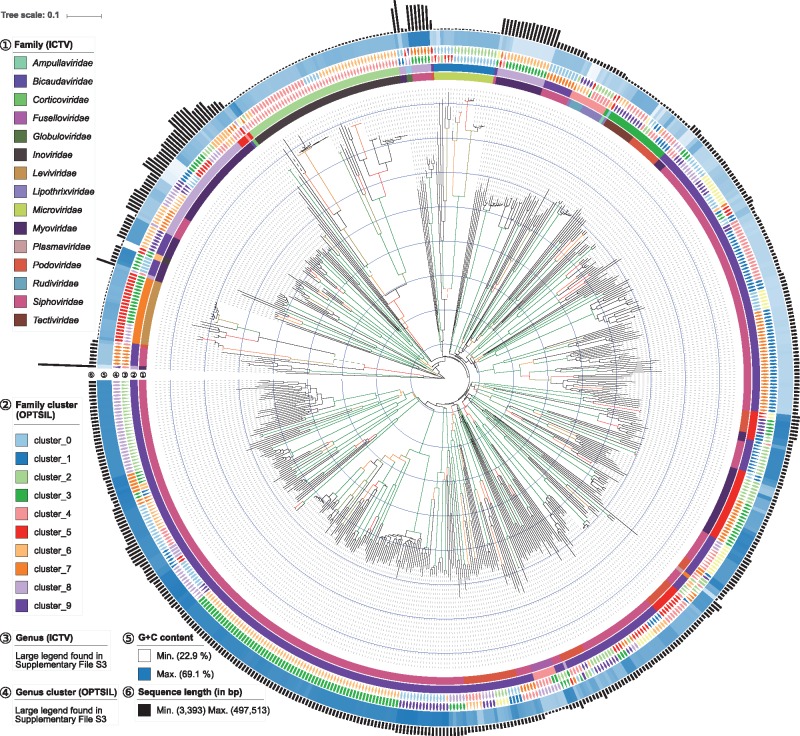
Phylogeny of prokaryotic viruses inferred from the amino-acid sequences contained in the reference dataset. Four dashed branches were downscaled by a factor of four to ease visualization of the tree. Genome size, genomic G + C content, ICTV genera and families as well as clusters derived from the ICTV genera and families are shown next to the tips within circles 1–6. For both the tree and the clusterings the optimal GBDP settings (Table 1) were used. Branch support ≥ 60% is shown as a colour gradient from red to green; terminal branches and branches with support < 60% are black. A linear visualization is provided in Supplement File S3 (Color version of this figure is available at *Bioinformatics* online.)

**Fig. 3.**
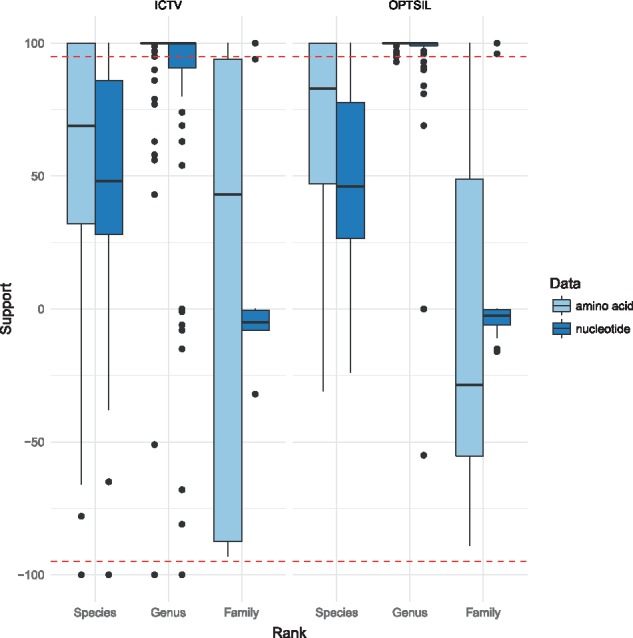
Taxon support at each taxonomic rank for ICTV taxa and clusters derived from these taxa. The underlying phylogenies and clusterings are based on the optimal GBDP settings provided in Table 1 for nucleotide and amino-acid sequences, the clusterings also on the according distance thresholds *T*. The dashed lines indicate areas of significant support (≥95%) or conflict ( ≤−95%); taxon support is displayed as negative in the case of non-monophyletic taxa (Color version of this figure is available at *Bioinformatics* online.)

Figure 2 shows the phylogenomic tree inferred from the amino-acid sequences under the optimal GBDP settings (Table 1). It is also contained in Supplementary File S3 in linear shape to allow for a more detailed analysis; the according nucleotide tree is contained in Supplementary File S4. Families did not usually appear as monophyletic, but did not induce significant conflict either, due to the low resolution of the backbone of the tree, especially in the case of the nucleotide sequences. In contrast, particularly the genera were highly supported as monophyletic with only few exceptions (Supplementary File S1). The distribution of the positive and negative support values for each taxon (Fig. 3) indeed indicated only a handful of well-supported conflicts. These were uniformly due to problematic assignments of names rather than due to the GBDP tree.

For instance, genus *L5likevirus* was supported as non-monophyletic with 100% support because of the positioning of *Mycobacterium phage Ta17a*. Genome sizes and G + C content values confirm that *Ta17a*, which was not included in the original description of *L5likevirus* (Hatfull and Sarkis, 1993), deviates from the majority of the representatives of the genus and thus was most likely later on incorrectly assigned to that taxon, or the genome KF024722 incorrectly assigned to that species. KF024722 is indeed nearly identical to the *Mycobacterium phage rosebush* (genus *Pgonelikevirus*) genome AY129334 and maximally supported as its sister group (Supplementary File S3).

Similarly, the species *Enterobacteria phage T5* appears as non-monophyletic in the tree because of the positioning of *Enterobacteria phage T5* (AY509815) (Supplementary Files S1 and S3). Its genome size clearly deviates from the main group of three T5 genomes, caused by its incomplete and aberrantly annotated genome sequence. The GBDP formulas selected as optimal (Table 1) presuppose complete genomes, whereas, e.g. formula *d*_4_ is robust (Meier-Kolthoff *et al.*, 2013) against the use of incomplete genomes and placed all four T5 genomes in a well-supported monophyletic group (data not shown).

### 3.3 Clusters versus classification

The tree includes groups of very closely related virus species that would form a single species according to the optimal species delineation threshold, such as the nearly identical *Enterobacteria phage f1*, *fd* and *M13*; *Caulobacter phage karma*, *magneto*, *phicbk* and *swift*; *Mycobacterium phage arturo*, *backyardigan*, *LHTSCC* and *peaches*, *Pseudomonas phage 14-1*, *F8*, *LBL3*, *LMA2*, *PB1*, and *SN*; and *Staphylococcus phage K* and *G* (Supplementary File S3). Whereas these clusters corresponded to reasonably to maximally (83, 100, 100, 100 and 100%) supported clades, branch support for the assigned species was lacking, probably because of the high uniformity of the genomes within the clusters. Conversely, a number of species were suggested to be split. For instance, *Vibrio phage CTX* was distributed over four clusters, in agreement with differences in genome size, whereas *Enterobacteria phage P1* and *Enterobacteria phage Qbeta* were split into two clusters, respectively. Dissecting such species did not necessarily yield higher branch support though.

Overall, however, taxon support for the taxon-derived clusters was considerably higher than for the taxa themselves at the genus and species rank when inferred from amino-acid sequences (Table 1; Fig. 3). A similar but weaker effect was observed for the nucleotide sequences, whereas no improvement was observed at the level of families. This not only indicated that deviations from ultrametricity hardly present a problem for the selected methods but also that, except for the families, from a phylogenetic viewpoint the classification would benefit from modifications that yielded taxa of more uniform genetic divergence. The MRI value for the genera was considerably larger than for the species, hence more effort would be needed to transform the species into groups of comparable divergence (Table 1).

### 3.4 Delineation of taxa at the subfamily level

Based on the optimal GBDP settings (Table 1) and the dataset reduced to 87 genomes, the taxon support at the ICTV subfamily level was 1 (amino acid) and 0 (nucleotide), respectively, for ICTV taxa as well as optimal OPTSIL clusters. High MRI values were observed, particularly for amino acids (0.97; nucleotides, 0.84). The subfamily delineation threshold for the nucleotide data (0.997270) was only marginally lower than the one for the families, whereas the subfamily delineation threshold for the amino-acid data (0.888940) did not collide with the genus and family thresholds. The resulting 76 OPTSIL subfamily clusters are annotated in Supplementary File S3.

### 3.5 Analysis of the expanded prokaryotic virus dataset

The comprehensive dataset contained 4419 genomes, which amounted to 9 765 990 pairwise comparisons. Supplementary File S5 shows the phylogenomic tree inferred from the amino-acid sequences of the 4K genomes under the best GBDP setting. The application of the optimal *T* values and the OPTSIL programme yielded an assignment into 49 families, 369 subfamilies, 721 genera and 2511 species. These differences from the reference dataset (Table 1) are as expected given the c. seven times larger expanded dataset. The distribution of taxon support in the expanded dataset (Supplementary File S5) was similar to the one for the reference dataset (Fig. 3). A total of 2105 new species clusters (84%), i.e. clusters not covering any species listed in the ICTV classification, 598 new genus clusters (83%), 288 new subfamily clusters (78%) and 28 new family clusters (57%) were found. The three largest new species clusters comprised mainly *Enterobacteria phage phiX174* (98 genomes), mainly *Propionibacterium phage* (88 genomes) and mainly *Synechococcus phage ACG-2014d* (45 genomes), respectively.

The plots of the host specificity in Supplementary File S6 (panel A) do not indicate a marked difference between the ICTV classification, the clustering of the reference dataset and the clustering of the expanded dataset. Most species of prokaryotic viruses appeared to be specific at the level of host species, and there was no overall trend of decreasing host specificity with increased sampling. Virus species not specific for a single host species was mostly specific for a host genus and, with a single exception, specific for a host family (Supplementary File S6, panel B). Entire virus genera were not in general specific for host species, and even specificity for host genera decreased for better sampled taxa; as a rule, virus genera were specific for host families. Virus families did not display any specificity that was independent of sampling (Supplementary File S6, panel B).

## 4 Discussion

### 4.1 GBDP for classification and phylogeny of bacterial and archaeal viruses

Our results clearly indicate that phylogeny and classification of prokaryotic viruses with GBDP is feasible. Except for the family rank, the selected settings yielded reasonably supported trees that agreed well with G + C content values and genome sizes (Fig. 2). Only a handful of significantly supported conflicts with the ICTV classification remained, which were uniformly due to misclassifications or incorrectly annotated GenBank sequences. This success might be due to the great adaptability of GBDP to solving specific phylogenomic questions. Well-performing settings can be chosen from a large number of distinct combinations of GBDP parameters, which appear to cover a range of options comparable to, if not exceeding, the range of settings previously investigated in the virus literature (Supplementary File S1).

Moreover, the optimized clustering approaches, even though they are not proper phylogenetic methods and are potentially affected by deviations from ultrametricity, here yielded higher taxon support than the original ICTV taxa, particularly at the genus rank and at the species level in the case of amino-acid sequences. This holds even though the inferred virus phylogeny partially strongly deviates from ultrametricity (Fig. 2). Whereas earlier studies (Krupovic *et al.*, 2016) reported ‘spurious taxonomic lumping’ when applying (potentially non-optimal) clustering methods to prokaryotic virus genomes, we observed on average higher branch support for the clusters compared with the original ICTV taxa. Thus parts of the ICTV classification could be improved phylogenetically, too, by generating some virus species and genera more uniform in terms of sequence divergence. Whereas we believe that this would ease the interpretation of virus classification as in the case of bacterial species (Auch *et al.*, 2010a; Meier-Kolthoff *et al.*, 2013) and would not affect the host specificity of the resulting taxa (Supplementary File S6), our primary criterion was phylogenetic support for the current taxa. It is up to the virus taxonomists to which extent sequence divergence should be used as secondary criterion, but augmenting GBDP phylogenies with clustering results is likely to assist in delineating viral taxa. The ideal classification would then only contain phylogenetically well-supported taxa displaying a sequence divergence within the range typical for their rank. Apparently such a classification could realistically be obtained under optimal GBDP settings at the subfamily, species and genus level.

Distinct structural annotations of a virus genome might yield distinct numbers of protein sequences. A nucleotide sequence of a phage might even represent overlapping genes and code for multiple proteins (Chirico *et al.*, 2010). The optimal GBDP settings include formulas *d*_0_ or *d*_6_ (Table 1), which consider gene content (Meier-Kolthoff *et al.*, 2013), and could thus be vulnerable against differences in protein composition due to distinct annotation. However, part of these issues might already be removed by the culling of paralogous sequences conducted by GBDP (Henz *et al.*, 2005), and we did not observe any apparently annotation-related issues in our analyses (Fig. 2). Moreover, GBDP works well with nucleotide sequences at the species and genus level (Fig. 3), which helps avoiding annotation artefacts entirely.

Phages are known to be affected by horizontal gene transfer (HGT), and, particularly when occupying similar ecological niches, this can lead to a high degree of mosaic diversity (Hendrix, 2003). However, the extent of HGT varies between different virus families and only partially affects certain viral functions (Krupovic *et al.*, 2011). Whereas some authors have concluded that hierarchical classification should rather not be aimed at in such situations (Hatfull, 2008; Lima-Mendez *et al.*, 2008; Nelson, 2004), we believe that as in the case of *Archaea* and *Bacteria*, the Linnaean hierarchy is feasible and useful despite a prevalence of HGT (Klenk and Göker, 2010). Rather, phylogenetic inference should ensure that branch support reflects the proportion of genes in agreement with that branch (Simon *et al.*, 2017). A taxonomic classification that assigned taxa only to well-supported clades could then hardly be called into question because of HGT. The partition bootstrap, which bootstraps entire genes instead of single positions in concatenated gene alignments, was suggested to reduce conflict and to provide more realistic support values in phylogenomic analyses (Siddall, 2010). In contrast, in ordinary bootstrapping, longer genes have a proportionally higher impact than shorter genes. The here selected best GBDP methods use pseudo-bootstrapping in conjunction with the greedy-with-trimming algorithm (Meier-Kolthoff *et al.*, 2014b), which is as close as possible within the GBDP framework to the partition bootstrap (Hahnke *et al.*, 2016). Hence, a strong GBDP pseudo-bootstrap value for a branch indicates that it is supported by at least the majority of the genes.

Although a common evolutionary origin of *Caudovirales* has been proposed (Veesler and Cambillau, 2011), and further studies suggested that most of the genes of contemporary phages derive from a common ancestral pool of genes (Hendrix *et al.*, 1999), multiple origins of phages might argue against a phylogenetic framework for their classification and even against their comprehensive phylogenetic analysis (Fig. 2). However, a carefully chosen method for the inference of trees, when applied to several lineages of independent origin, should simply leave their relative positioning, i.e. the backbone of the tree, unresolved. A taxonomic classification focusing on well-supported branches could then hardly be affected by the independent origin of the major lineages.

Lack of support at the backbone of the phylogenetic tree (Fig. 2) and for the virus families (Fig. 3) was indeed observed for the current dataset, and could not be solved by switching to clustering approaches optimized for the family rank. Rampant early HGT and multiple origins of prokaryotic viruses are possible causes of this lack of resolution. Still stronger support for the families (and subfamilies) at the amino-acid level rather than the nucleotide level (Fig. 3) alternatively points to a fading of the phylogenetic signal due to the rapid evolution or an ancient origin of prokaryotic viruses (Hatfull, 2008), in line with the fact that affiliation of viruses to the same family is currently possible without any DNA sequence relatedness (Nelson, 2004). Within the current GBDP framework, this issue could only be solved by creating less divergent families. We do not think such a step is necessary, however, since the assignment of prokaryotic viruses to families is currently mainly based on characteristics such as morphology, replication mode and overall genomic architecture (Hendrix *et al.*, 1999); some of these features, such as neck organization of tailed phages, can even be inferred from sequences (Lopes *et al.*, 2014). Conversely, convergent evolution can explain the resemblance between apparently unrelated viruses (Ackermann, 2007), and we cannot rule out that some of the character states used to define virus families are plesiomorphic or homoplasious (Farris, 1974; Hennig, 1965; Wiley and Lieberman, 2011) and in conflict with sequence data (Nelson, 2004). However, GBDP is not suggested as a replacement for these features but for solving questions their analysis cannot address.

Taxa at the currently hardly applied subfamily level can now be delineated using GBDP in conjunction with amino-acid sequences, which will most likely result in phylogenomically well supported groups of genera below the level of the family. Indeed, amino acid-based subfamily delineation yielded high taxon support values for both ICTV taxa and OPTSIL clusters, high MRI values and a threshold placed between genus and family boundaries and well part from either. In contrast, subfamily delineation with GBDP and nucleotide sequences cannot currently be recommended.

The analysis of the expanded dataset demonstrated that the optimized GBDP settings can be used to classify prokaryotic viruses at the level of species and genera, even those not yet listed in the ICTV taxonomy. We did not observe significant differences to the reference dataset in terms of the quantitative behaviour of the taxa at the distinct ranks. Rather, the vast majority of potential taxa are simply not yet covered by the ICTV classification (Roux *et al.*, 2015). We thus expect an increased taxonomic coverage to yield little conflict but new insights due to the sheer amount of data.

For instance, the comparison of the reference and the expanded dataset indicated that the specificity of virus species for host species and that of virus genera for host families is not an artefact of insufficient sampling but rather a real feature of the data. Switching from the ICTV taxa to OPTSIL clusters (derived from these taxa) and increasing the genome sampling to the expanded dataset did not change these patterns. Broad host ranges were observed, for instance, in the case of phages whose adsorption to the host cell walls depends on the presence of certain plasmids (Olsen *et al.*, 1974). Low host specificity might also be an adaptation to low concentrations of host cells (Chibani-Chennoufi *et al.*, 2004). However, viruses observed in the laboratory are mostly specific to host species, and the analysis of marine phages even indicates strain specificity in some cases (Chibani-Chennoufi *et al.*, 2004). Even though, within the scope of this study, we could not account for a possibly still biased sampling, for wrongly assigned phage hosts and for prokaryotic taxa that do not reflect the phylogenetic relationships of the hosts, the results on host specificity thus appear entirely reasonable.

### 4.2 Publicly available web service

The best combination of GBDP parameters determined in this study for phylogenetic inference from whole nucleotide and proteome sequences of prokaryotic viruses were incorporated into the standalone web service ‘VICTOR’, the VIrus Classification and Tree building Online Resource. Its results include phylogenomic trees with branch support and augmented with suggestions for taxon boundaries, thus allowing for an informed taxonomic decision. Users can specify sequences of currently up to 100 viruses in several file formats as well as indicate whether nucleotide or amino-acid sequences should be analysed. When complete genome sequences are missing for some viruses of interest, users can refrain from considering the optimal formulas *d*_0_ or *d*_6_ (Table 1) and use the formula *d*_4_ instead, which is not the best performing formula when applied to completely sequenced genomes and thus not generally recommended but robust against the use of incomplete genome sequences (Meier-Kolthoff *et al.*, 2013).

The web service is available as a rapid yet reliable bioinformatics application free of charge at https://victor.dsmz.de. Whereas it was beyond the scope of our study to also examine viruses of plants and animals, we see no reason why the service should not be used to elucidate their phylogeny as well; determining how to best delineate their taxa with GBDP is a logical next step.

## Supplementary Material

Supplementary Data
