# Supplementary material for: VICTOR: genome-based phylogeny and classification of prokaryotic viruses
Source: Bioinformatics. 2017 Jul 7;33(21):3396–404. doi: 10.1093/bioinformatics/btx440 (PMC5860169; doi:10.1093/bioinformatics/btx440)
Supplement: Supplementary Data [file btx440_supp.zip › supplement_description.pdf]

VICTOR: Genome-based Phylogeny and Classification of Prokaryotic Viruses

Jan P. Meier-Kolthoff[1,*] and Markus Göker[1]

[1] Leibniz Institute DSMZ – German Collection of Microorganisms and Cell Cultures, Inhoffenstrasse 7B, 38124 Braunschweig, Germany

* To whom correspondence should be addressed. Email: jan.meier-kolthoff@dsmz.de

Description of Supplementary Material

Supplementary File S1 – First sheet, list of INSDC accessions of (annotated) genome sequence that were successfully mapped to a phage species from the ICTV master table, G+C content values, genome sizes, complete ICTV annotation and cluster affiliation under the selected GBDP settings. Second sheet, taxon support and MRI values obtained for all 240 examined GBDP settings. Third sheet, phylogenetic results for ICTV taxa obtained using amino-acid sequences, assessed under the respective optimal GBDP settings. Fourth sheet, phylogenetic results for ICTV taxa obtained using nucleotide sequences, also assessed under the respective optimal GBDP settings. Fifth sheet, phylogenetic results for OPTSIL clusters inferred from the amino-acid sequences. Sixth sheet, phylogenetic results for OPTSIL clusters inferred from the nucleotide sequences. Seventh sheet, list of phage genomes within the expanded data set, G+C content values, genome sizes and cluster affiliation under the selected GBDP settings. Eighth sheet, cross-comparison of tools for the sequence-based comparison of phages, illustrating the specifics and limitations of each methods.

Supplementary File S2 – R code used for the statistical analyses, given file S1.

Supplementary Table S3 – Phylogenomic tree in linear representation inferred from the amino-acid sequences of the reference data set under optimal GBDP settings.

Supplementary File S4 – Phylogenomic tree inferred from the nucleotide sequences of the reference data set under optimal GBDP settings.

Supplementary File S5 – Phylogenomic tree inferred from the amino-acid sequences of the expanded data set under optimal GBDP settings. Also contains a plot of the taxon support of the inferred clusters at each rank.

Supplementary File S6 – Host specificity at all taxonomic ranks. Panel A shows the distribution of the specificity at each of the three taxonomic ranks investigated for host taxa on the one hand and ICTV taxa as well as clusters derived from these taxa (Table 1) on the other hand. Panel B shows the host specificity additionally in dependence on *N*, the overall number of phages with an interpretable host entry per taxon or cluster, as visualized using separate robust-line fits for each subset of the data. Clusters were calculated from both the reference (ICTV) and the expanded (4K) data set.
