# Supplementary material for: VICTOR: genome-based phylogeny and classification of prokaryotic viruses
Source: Bioinformatics. 2017 Jul 7;33(21):3396–404. doi: 10.1093/bioinformatics/btx440 (PMC5860169; doi:10.1093/bioinformatics/btx440)
Supplement: Supplementary Data [file btx440_supp.zip › S2_misc_code_and_settings_for_reproducible_science.pdf]

Supplementary File S2

Article reference

Article: VICTOR: Genome-based Phylogeny and Classification of Prokaryotic Viruses

Authors: Jan P. Meier-Kolthoff and Markus Göker

Affiliation: Leibniz Institute DSMZ – German Collection of Microorganisms and Cell Cultures

Table of contents

This file contains all the R source code written to analyse and produce the figure 1, 3 and 4 shown in this manuscript. ## R code used for generation of main manuscript Figure 1 Cleaning the R workspace

```
rm(list = ls(all = TRUE))
```

```
library(devtools)
```

```
##
## Attaching package: 'devtools'

## The following object is masked from 'package:pkgutils':
##
##     check
```

```
library("FactoMineR") # install via: install_github("kassambara/factoextra")
library("factoextra")
```

```
## Loading required package: ggplot2
```

```
library(reshape2)
```

loading data ...

```
ds.all.criteria <- read.table("data/supplementary_file_S1__second_sheet.csv",
                              sep="\t", header=T)

step.width = 0.05

cols.of.interest <- c("GBDP_combo", "Data_type",
                      "Average_support",
                      "taxon.support.Rank.Species",
                      "taxon.support.Rank.Genus",
                      "taxon.support.Rank.Family")
```

defining relevant column names ...

```
cols.of.interest <- c(cols.of.interest,
                      paste("MRI.for.F", seq(0,1,step.width),
                            ".orig_species_ref_part", sep=""),
                      paste("MRI.for.F", seq(0,1,step.width),
                            ".orig_genus_ref_part", sep=""),
                      paste("MRI.for.F", seq(0,1,step.width),
                            ".orig_family_ref_part", sep=""))
```

```r
ds.all.criteria.small.full <- ds.all.criteria[,names(ds.all.criteria) %in% cols.of.interest]

ds.all.criteria.small <- ds.all.criteria.small.full

cols.for.biplot <- c("taxon.support.Rank.Family",
                     "taxon.support.Rank.Genus",
                     "taxon.support.Rank.Species",
                     "MRI.for.F0.5.orig_species_ref_part",
                     "MRI.for.F0.5.orig_genus_ref_part",
                     "MRI.for.F0.5.orig_family_ref_part")

pretty.col.names <- c("support (family)",
                      "support (genus)",
                      "support (species)",
                      "MRI (species)",
                      "MRI (genus)",
                      "MRI (family)")
```

dataframe for biplot

```r
ds.phage <- ds.all.criteria.small[,colnames(ds.all.criteria.small) %in% cols.for.biplot]

colnames(ds.phage) <- pretty.col.names

data.type <- ds.all.criteria.small[,2]
```

splitting the strings and using "unlist" to get rid of list type

```r
splitted.method.col <- unlist(strsplit(as.character(ds.all.criteria.small$GBDP_combo), "_"))
```

problem: "unlist" yields a huge character vector with all the splitted items as one big chain solution: we need to tell R to forge a matrix from it, by exactly providing the dimensions (you can think of it as a line wrap)

```r
splitted.method.col <- matrix(splitted.method.col, ncol=6, nrow=360, byrow=T)

ds.all.criteria.small <- cbind(splitted.method.col, ds.all.criteria.small)

algo <- ds.all.criteria.small$`5`
formula <- ds.all.criteria.small$`6`
```

Getting principal components ...

```r
#
```

apply PCA - scale. = TRUE is highly advisable, but default is FALSE.

```r
#
phage.pca <- prcomp(ds.phage,
                    center = TRUE,
                    scale. = TRUE)
```

rotation: the matrix of variable loadings (i.e., a matrix whose columns contain the eigenvectors). The function princomp returns this in the element loadings.

```r
phage.pca$rotation
```

```
##                         PC1         PC2         PC3         PC4        PC5
## support (family)  0.3940784 -0.19394310  0.65928540  0.42227055 -0.3895048
## support (genus)   0.4842925  0.10239733  0.21011340 -0.14422546  0.2139273
```

```
## support (species) 0.4477513  0.14556846 -0.07307068 -0.73381438 -0.3800019
## MRI (species)     0.2543491 -0.78399943 -0.50583096  0.08859887 -0.1892544
## MRI (genus)       0.4772976 -0.06317524 -0.05594920  0.08181668  0.7459385
## MRI (family)      0.3423087  0.55862731 -0.50681410  0.49785473 -0.2566676
##                          PC6
## support (family)   0.20592393
## support (genus)   -0.80265936
## support (species)  0.30017370
## MRI (species)     -0.14532544
## MRI (genus)        0.44938560
## MRI (family)      -0.01273326
```

report importance of components:

```r
summary(phage.pca)
```

```
## Importance of components:
##                           PC1    PC2    PC3     PC4     PC5    PC6
## Standard deviation     1.9577 0.9683 0.8029 0.56958 0.43251 0.2717
## Proportion of Variance 0.6387 0.1563 0.1074 0.05407 0.03118 0.0123
## Cumulative Proportion  0.6387 0.7950 0.9024 0.95652 0.98770 1.0000
```

We can use the predict function if we observe new data and want to predict their PCs values. Just for illustration we can pretend the last two rows of the phage data are "new" and we want to see what is their PCs values:

```r
predict(phage.pca, newdata=tail(ds.phage, 2))
```

```
##           PC1       PC2       PC3        PC4        PC5       PC6
## 359 0.8583184 0.4309741 0.8090312 -0.5857897  0.3871558 0.2071767
## 360 1.6010788 0.6364654 1.1099306 -0.1303815 -0.3111733 0.4123755
```

Drawing the actual biplot:

A biplot helps to interpret the factorial axes while looking at the individual obs. location. The loading of each variable (i.e., different MRis and taxon.support values) on the first two principal components. The principal components are pointing in the most-varying direction of the data. The data is plotted on the rotated scale, and thus the horizontal axis is the most-varying direction of the data. The arrows are pointing in the direction of the variables, as projected into the 2-d plane of the biplot. The first principal axis is the one which maximizes the variance (reflected by its eigenvalue). The second one is orthogonal to it, and still maximizes the remaining variance. performs Principal Component Analysis using PCA from the FactoMineR package:

```r
p.pca <- PCA(ds.phage, graph = FALSE)
```

PCA Biplot (with cos2, i.e., quality of the representation for variables on the factor map). The squared loadings for variables are called cos2 (= cor $cor = coord$ coord). The sum of the cos2 for variables on the principal components is equal to one. If a variable is perfectly represented by only two components, the sum of the cos2 is equal to one. In this case the variables will be positioned on the circle of correlations. For some of the variables, more than 2 components are required to perfectly represent the data. In this case the variables are positioned inside the circle of correlations. The squared loadings for variables (cos2; if a variable is perfectly represented by only two components, the sum of the cos2 is equal to one):

```r
head(p.pca$var$cos2)
```

```
##                       Dim.1       Dim.2       Dim.3       Dim.4
## support (family)  0.5951668 0.035267749 0.280206092 0.057849195
## support (genus)   0.8988537 0.009831195 0.028460214 0.006748381
## support (species) 0.7683291 0.019868434 0.003442048 0.174698092
```

```
## MRI (species)     0.2479328 0.576315843 0.164945876 0.002546667
## MRI (genus)       0.8730760 0.003742164 0.002017988 0.002171698
## MRI (family)      0.4490653 0.292599371 0.165587682 0.080412025
##                        Dim.5
## support (family)  0.028380735
## support (genus)   0.008561118
## support (species) 0.027012800
## MRI (species)     0.006700233
## MRI (genus)       0.104088804
## MRI (family)      0.012323674
```

Correspondence analysis (CA) is an extension of Principal Component Analysis (PCA) suited to analyze frequencies formed by two categorical variables. fviz_ca() provides ggplot2-based elegant visualization of CA outputs from the R functions:

```r
fviz_ca_row(p.pca) + labs(title = "", x = "PC1", y ="PC2" )
```

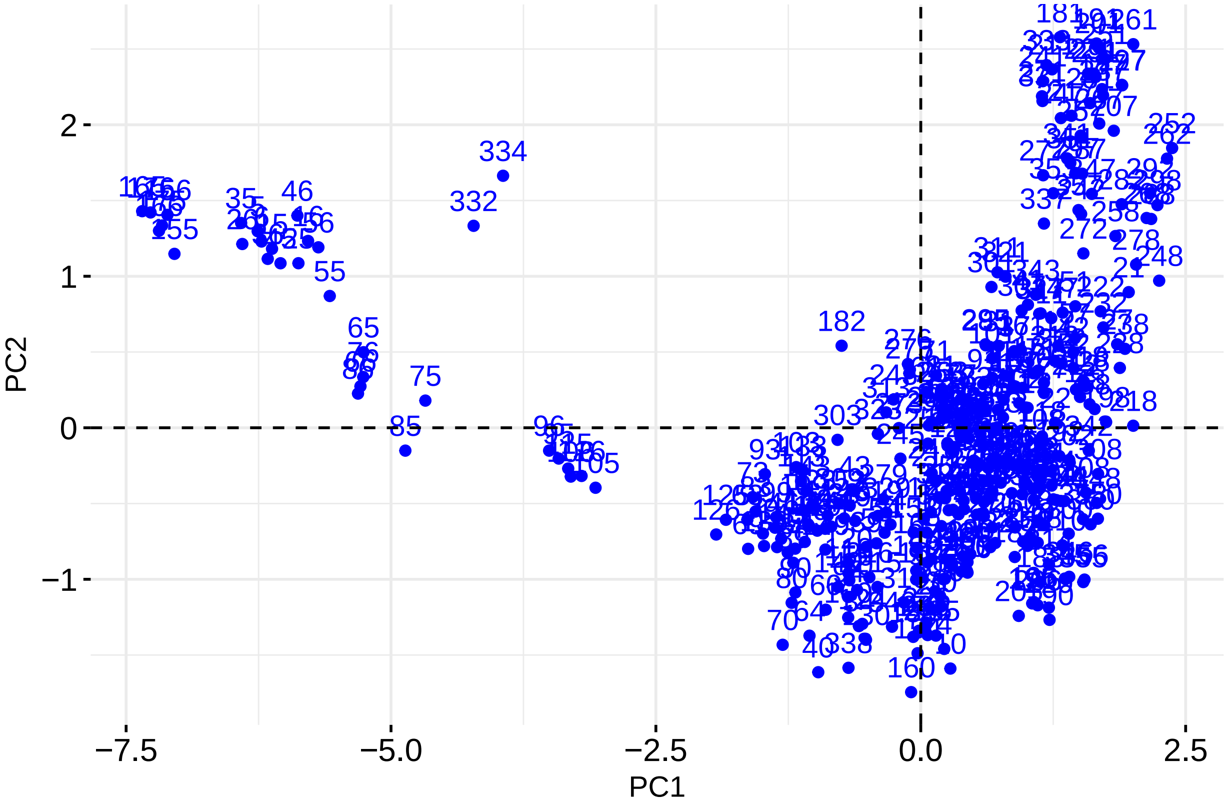

Principal component analysis (PCA) reduces the dimensionality of multivariate data, to two or three that can be visualized graphically with minimal loss of information. Here we reduce to two dimensions (biplot).

```r
p <- fviz_pca_biplot(p.pca,
                     habillage = data.type,
                     addEllipses = TRUE,
                     ellipse.level = 0.95,
                     palette="Dark2",
                     legend.partial.title = "gulp",
                     circle = T,
                     col.var = "blue",
                     col.circle = "black",
                     label = "var") +
```

```r
  labs(title = "",
       x = paste("\nPC1 (", round(p.pca$eig[1,2],1), "% explained var.)",  sep=""),
       y = paste("PC2 (", round(p.pca$eig[2,2],1), "% explained var.)\n",  sep="")) +
  theme_minimal()

print(p)
```

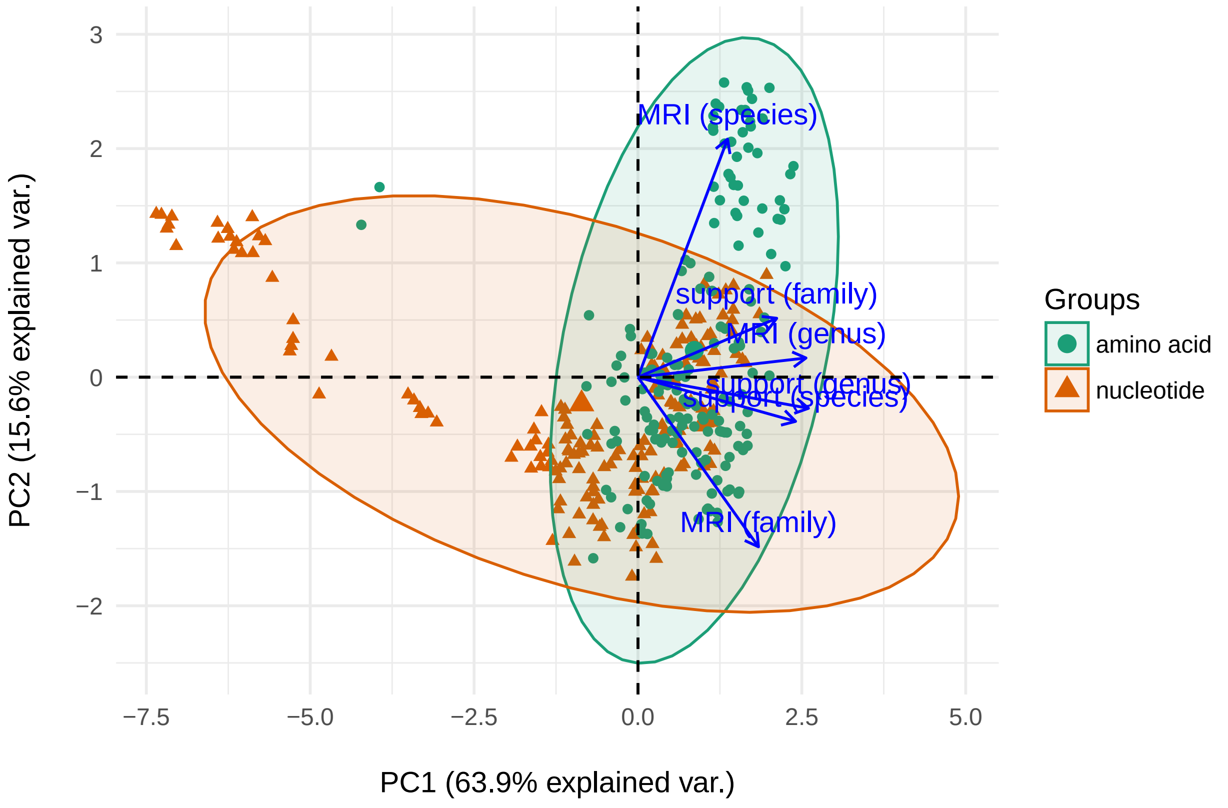

R code used for generation of main manuscript Figure 3

cleaning the R workspace

```r
rm(list = ls(all = TRUE))
```

```r
library(ggplot2)
library(plyr)
library(MASS)
```

loading phylogenetic results (with regard to the **ICTV classification**) for individual taxa obtained using **amino-acid** sequences, assessed under the respective optimal GBDP settings

```r
df.mash.prot <- read.table("data/supplementary_file_S1__third_sheet.csv",
                           sep="\t",
                           header=T)

df.mash.prot$data.type <- rep("amino acid", nrow(df.mash.prot))
df.mash.prot$classif.type <- rep("ICTV", nrow(df.mash.prot))
```

loading phylogenetic results (with regard to the **ICTV classification**) for individual taxa obtained using **nucleotide** sequences, assessed under the respective optimal GBDP settings

```
df.mash.nuc <- read.table("data/supplementary_file_S1__fourth_sheet.csv",
                          sep="\t",
                          header=T)

df.mash.nuc$data.type <- rep("nucleotide", nrow(df.mash.nuc))
df.mash.nuc$classif.type <- rep("ICTV", nrow(df.mash.nuc))
```

loading phylogenetic results (with regard to the **OPTSIL clustering**) for individual taxa obtained using **amino-acid** sequences, assessed under the respective optimal GBDP settings

```
df.OPTSIL.classif.prot <- read.table("data/supplementary_file_S1__fifth_sheet.csv",
                                     sep="\t",
                                     header=T)

df.OPTSIL.classif.prot$data.type <- rep("amino acid", nrow(df.OPTSIL.classif.prot))
df.OPTSIL.classif.prot$classif.type <- rep("OPTSIL", nrow(df.OPTSIL.classif.prot))
```

loading phylogenetic results (with regard to the **OPTSIL clustering**) for individual taxa obtained using **nucleotide** sequences, assessed under the respective optimal GBDP settings

```
df.OPTSIL.classif.nuc <- read.table("data/supplementary_file_S1__sixth_sheet.csv",
                                    sep="\t",
                                    header=T)

df.OPTSIL.classif.nuc$data.type <- rep("nucleotide", nrow(df.OPTSIL.classif.nuc))
df.OPTSIL.classif.nuc$classif.type <- rep("OPTSIL", nrow(df.OPTSIL.classif.nuc))
```

merge the four different input data frames

```
df.all <- rbind(df.mash.nuc, df.mash.prot,
                df.OPTSIL.classif.nuc, df.OPTSIL.classif.prot)
```

filter out "trivial" taxa ...

```
idx <- which(!df.all$Status == "trivial")
df.all <- df.all[idx,]
```

creating support column 'support.for.plot' for boxplot: support of non-monophyletic taxa is negated

```
df.all$support.for.plot <- ifelse(df.all$Status == "monophyletic",
                                  df.all$Max.support,
                                  -df.all$Max.support)
```

reorder levels to have boxplots of different ranks properly ordered from species to family

```
df.all$Rank <- factor(df.all$Rank, levels = c("Species",
                                              "Genus",
                                              "Family"))

p <- ggplot(df.all, aes(factor(Rank), support.for.plot)) +
  geom_boxplot(aes(fill = factor(data.type))) +
  xlab("\nRank") +
  ylab("Support\n") +
  scale_fill_brewer("Data", palette="Paired") +
  geom_hline(yintercept=95, colour = "red", linetype="dashed") +
  geom_hline(yintercept=-95, colour = "red", linetype="dashed") +
  theme_minimal() +
  facet_wrap(~classif.type)
```

```r
print(p)
```

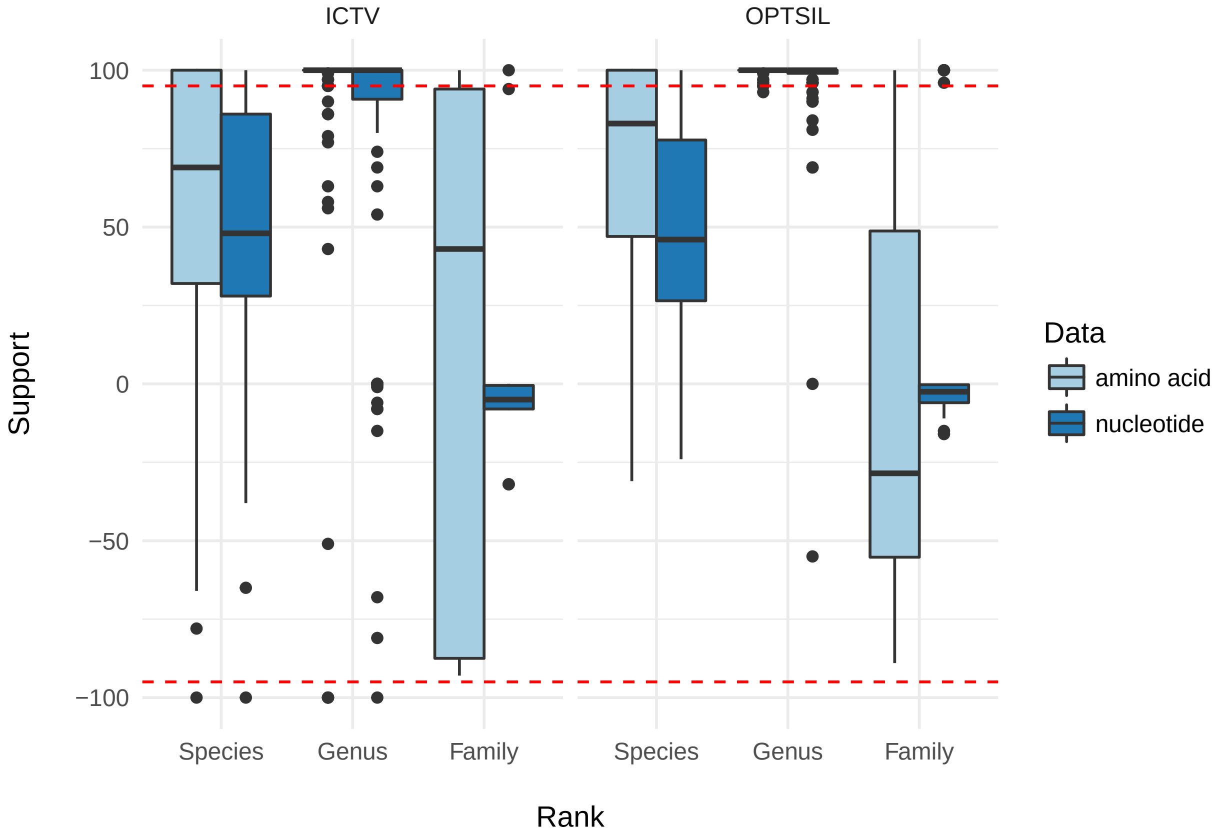

final summary showing the indiviudual means of the different distributions:

```r
aggregate(df.all$support.for.plot, list(df.all$Rank,
                                        df.all$data.type,
                                        df.all$classif.type), mean)
```

```
##    Group.1    Group.2 Group.3          x
## 1  Species amino acid    ICTV  51.306122
## 2    Genus amino acid    ICTV  87.062500
## 3   Family amino acid    ICTV   6.909091
## 4  Species nucleotide    ICTV  48.102041
## 5    Genus nucleotide    ICTV  75.937500
## 6   Family nucleotide    ICTV   8.909091
## 7  Species amino acid  OPTSIL  70.294737
## 8    Genus amino acid  OPTSIL  99.687500
## 9   Family amino acid  OPTSIL -10.200000
## 10 Species nucleotide  OPTSIL  49.322581
## 11   Genus nucleotide  OPTSIL  94.691358
## 12  Family nucleotide  OPTSIL  14.846154
```

R code used for generation of main manuscript Figure 4

cleaning the R workspace

```r
rm(list = ls(all = TRUE))
```

simple dictionary which enables user to easily change labels in final plots

```r
cluster_type_mapping <- c(
  `Phage species` = "Phage species",
  `Phage genus` = "Phage genus",
  `Phage family` = "Phage family"
)
```

simple dictionary which enables user to easily change labels in final plots

```r
host_mapping <- c(
  `host species` = "Host species",
  `host genus` = "Host genus",
  `host family` = "Host family"
)
```

later used in ggplot's facet_wrap layer

```r
global_labeller <- labeller(
  host.info.type = host_mapping,
  type = cluster_type_mapping,
  .default = label_both,
  .multi_line = F
)
```

Main function for calculating host homogeneity

@param df.data The input data frame @param host.field Name of the column storing the host information @param prefix A prefix which is appended to the output @param use.ICTV.classif.instead.of.OPTSIL Defines which clustering to use

```r
assess_host_homogeneity <- function(df.data,
                                    host.field,
                                    prefix,
                                    use.ICTV.classif.instead.of.OPTSIL){

  #' keep only entries with specific host
  idx <- which(df.data[,host.field] != "NA")
  df.data <- df.data[idx,]


  if(use.ICTV.classif.instead.of.OPTSIL){
    ranks.to.visit <- c("ICTV.species", "Genus", "Family")
  }else{
    ranks.to.visit <- c("Species", "Genus", "Family")
  }


  df.all <- data.frame()

  for(rank in ranks.to.visit){

    if(use.ICTV.classif.instead.of.OPTSIL){
      #' use ICTV species/genera/families as clusters ...
      df.rank <- df.data[, colnames(df.data) %in% c(rank)]
```

```
    df.rank <- df.data[c(rank, host.field)]
  }else{
    #' use the calculated OPTSIL cluster ...
    df.rank <- df.data[, colnames(df.data) %in% c(paste(rank, "_cluster_prot", sep=""),
                                                    host.field)]

    df.rank <- df.data[c(paste(rank, "_cluster_prot", sep=""),
                         host.field)]
  }

  colnames(df.rank) <- c("Cluster", "Host")
  categories <- unique(df.rank$Cluster)

  homogeneity_indices <- rep(NA, length(categories))
  Ns <- rep(NA, length(categories))
  ms <- rep(NA, length(categories))

  #' get proper type ...
  rank.display <- NA
  if(grepl("Species|ICTV.species", rank)){
    rank.display <- "Phage species"
  }else if(grepl("Genus", rank)){
    rank.display <- "Phage genus"
  }else{
    stopifnot(grepl("Family", rank))
    rank.display <- "Phage family"
  }
  type <- rep(rank.display, length(categories))
  df.all.tmp <- data.frame(type=type)

  for(i in 1:length(categories)){

    category <- categories[i]
    idx <- which(df.rank$Cluster == category)
    df.tmp <- df.rank[idx,]
    N <- nrow(df.tmp)
    #' determine the host which is most frequent and return this number
    m <- as.numeric(sort(table(df.tmp$Host), decreasing=TRUE)[1:1])

    #' index in analogy to Berger-Parker index (1970)
    homogeneity_idx <- m/N
    homogeneity_indices[i] <- homogeneity_idx
    Ns[i] <- N
    ms[i] <- m
  }

  df.all.tmp$categories <- as.character(categories)
  df.all.tmp$homogeneity_indices <- homogeneity_indices
  df.all.tmp$N <- Ns
  df.all.tmp$m <- ms

  df.all <- rbind(df.all,df.all.tmp)
}
```

```
  df.all$dataset <- rep(prefix,nrow(df.all))
  df.all$host.info.type <- rep(host.field,nrow(df.all))
  df.all$used.ICTV.classif.instead.of.OPTSIL <- rep(use.ICTV.classif.instead.of.OPTSIL,
                                                    nrow(df.all))

  return(df.all)
}
```

reading the input data

```
df.ictv <- read.table("data/supplementary_file_S1__first_sheet.csv",
                      sep="\t",
                      header=T)
```

keep only entries used for the phylogenetic study (610 phages) empty "remove" field indicates no problem with a particular phage

```
df.ictv <- df.ictv[which(df.ictv$Remove. == ""),]

df.4K <- read.table("data/supplementary_file_S1__seventh_sheet.csv",
                    sep="\t",
                    header=T,
                    quote = "\"'")
```

color 1:

```
df.all.1 <- assess_host_homogeneity(df.ictv,
                                    "Specific_host_species_checked", "ICTV / OPTSIL", F)

df.all.2 <- assess_host_homogeneity(df.ictv,
                                    "Specific_host_genus_checked", "ICTV / OPTSIL", F)

df.all.3 <- assess_host_homogeneity(df.ictv,
                                    "Specific_host_family_checked", "ICTV / OPTSIL", F)
```

color 2:

```
df.all.4 <- assess_host_homogeneity(df.4K,
                                    "Specific_host_species_checked", "4K / OPTSIL", F)

df.all.5 <- assess_host_homogeneity(df.4K,
                                    "Specific_host_genus_checked", "4K / OPTSIL", F)

df.all.6 <- assess_host_homogeneity(df.4K,
                                    "Specific_host_family_checked", "4K / OPTSIL", F)
```

color 3:

```
df.all.7 <- assess_host_homogeneity(df.ictv,
                                    "Specific_host_species_checked", "ICTV / ICTV", T)

df.all.8 <- assess_host_homogeneity(df.ictv,
                                    "Specific_host_genus_checked", "ICTV / ICTV", T)

df.all.9 <- assess_host_homogeneity(df.ictv,
                                    "Specific_host_family_checked", "ICTV / ICTV", T)
```

```
df.all <- rbind(df.all.1, df.all.2, df.all.3,
                df.all.4, df.all.5, df.all.6,
                df.all.7, df.all.8, df.all.9)
```

make factor ...

```
df.all$dataset <- factor( df.all$dataset)
```

reordering factor to get "ICTV" before "4K"

```
df.all$dataset <- factor( df.all$dataset, levels = c("ICTV / ICTV",
                                                     "ICTV / OPTSIL",
                                                     "4K / OPTSIL"))
```

nicer facet_wrap panel labels

```
df.all$host.info.type <- factor(df.all$host.info.type)
```

change ordering of factor levels ...

```
df.all$host.info.type <- factor(df.all$host.info.type,
                                levels = c("Specific_host_species_checked",
                                           "Specific_host_genus_checked",
                                           "Specific_host_family_checked"))


df.all$host.info.type <- revalue(df.all$host.info.type,
                                 c("Specific_host_species_checked"="host species",
                                   "Specific_host_genus_checked"="host genus",
                                   "Specific_host_family_checked"="host family"))

p.1 <- ggplot(df.all, aes(factor(type), homogeneity_indices, fill=dataset)) +
  geom_boxplot() +
  xlab("\nCluster") +
  ylab("Host specificity\n") +
  facet_wrap(~host.info.type, scales="free_y", ncol = 1, labeller = global_labeller) +
  scale_fill_brewer("Dataset / Classification", palette="Dark2") +
  theme_minimal() +
  theme(legend.position = "none")


p.2 <- ggplot(df.all, aes(N, homogeneity_indices)) +
  geom_point(size=1, aes(colour = dataset)) +
  ylab("Host specificity\n")  +
  xlab("\nN (total number of hosts per cluster)") +
  facet_wrap(host.info.type~type, scales="free_x", ncol=3, labeller = global_labeller) +
  scale_colour_brewer("Dataset / Classification", palette="Dark2") +
  coord_cartesian(ylim = c(0,1)) +
  theme_minimal() +
  theme(legend.position = "bottom",
        strip.text.x = element_text(size = 8, colour = "black", angle = 0)) +
  #' add robust line fit ...
  geom_smooth(size=1, aes(N, homogeneity_indices, colour=dataset), method=rlm,
              method.args = list(maxit=5000), fullrange=TRUE, level=0.95, se=F)
```

```
myplot <- grid.arrange(p.1,p.2, widths=c(0.36, 0.64), ncol=2)
```

```
## Warning in predict.lm(model, newdata = data.frame(x = xseq), se.fit = se, :
## prediction from a rank-deficient fit may be misleading
```

```
print(myplot)
```

```
## TableGrob (1 x 2) "arrange": 2 grobs
##   z     cells    name           grob
## 1 1 (1-1,1-1) arrange gtable[layout]
## 2 2 (1-1,2-2) arrange gtable[layout]
```

```
grid.text(x = unit(0.03, "npc"), y = unit(0.97, "npc"), label="A")
grid.text(x = unit(0.363, "npc"), y = unit(0.97, "npc"), label="B")
```

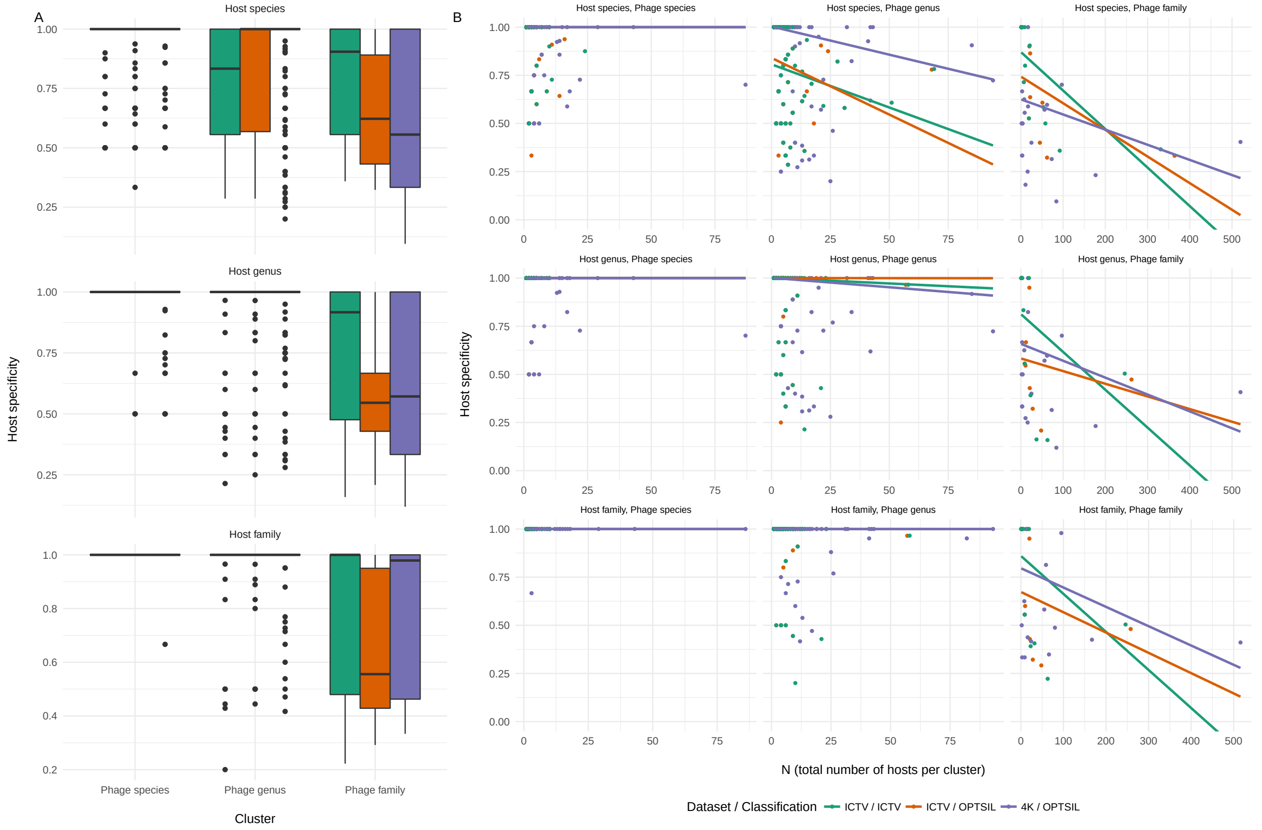

assess the Kendall correlations between homogeneity index and sampling size

```
all.host.types <- levels(df.all$host.info.type)
all.phage.types <- levels(df.all$type)
all.datasets <- levels(df.all$dataset)

num.correlations <- length(all.host.types) * length(all.phage.types) * length(all.datasets)

out.host <- rep(NA, num.correlations)
out.phage <- rep(NA, num.correlations)
out.dataset <- rep(NA, num.correlations)
out.kendall.tau <- rep(NA, num.correlations)
out.kendall.tau.p.value <- rep(NA, num.correlations)

l = 1
```

Note: we normally don't use such nested for-loops in R, rather one of the apply functions, but this part here

is not a performance bottleneck and the approach is a bite more intuitive to grasp that way

collecting all information regarding the Kendall correlation analysis

```
df.kendall <- data.frame(host = out.host,
                         phage_cluster = out.phage,
                         dataset = out.dataset,
                         kendall.tau = out.kendall.tau,
                         kendall.tau.p.value = out.kendall.tau.p.value)

idx <- which(df.all$dataset == "4K / OPTSIL" &
               df.all$host.info.type == "host family" &
               df.all$type == "Phage species" &
               df.all$homogeneity_indices != 1)

interesting.obs <- df.all[idx,]
```

example on how to reveal a potentially interesting species cluster via its cluster number (here: 2268)

```
idx.2 <- which(df.4K$Species_cluster_prot == "cluster_2268")
relevant.cluster <- df.4K[idx.2,]

options(width=120) # increase width to allow printing of all columns next to each other
print(df.kendall)
```

```
##             host phage_cluster       dataset kendall.tau kendall.tau.p.value
## 1  host species Phage species   ICTV / ICTV -0.60781722        4.751693e-42
## 2  host species Phage species ICTV / OPTSIL -0.58952191        9.622847e-35
## 3  host species Phage species   4K / OPTSIL -0.35065990        4.584941e-20
## 4  host species   Phage genus   ICTV / ICTV -0.33233956        1.709025e-05
## 5  host species   Phage genus ICTV / OPTSIL -0.42257268        9.365107e-09
## 6  host species   Phage genus   4K / OPTSIL -0.48255438        4.365122e-18
## 7  host species  Phage family   ICTV / ICTV -0.76761031        2.104331e-04
## 8  host species  Phage family ICTV / OPTSIL -0.70454545        5.191201e-03
## 9  host species  Phage family   4K / OPTSIL -0.40219075        3.857277e-03
## 10   host genus Phage species   ICTV / ICTV          NA                  NA
## 11   host genus Phage species ICTV / OPTSIL -0.18701962        1.203725e-03
## 12   host genus Phage species   4K / OPTSIL -0.28956073        4.079688e-14
## 13   host genus   Phage genus   ICTV / ICTV -0.31222533        4.795927e-04
## 14   host genus   Phage genus ICTV / OPTSIL -0.33349715        1.113942e-04
## 15   host genus   Phage genus   4K / OPTSIL -0.44248369        5.805057e-15
## 16   host genus  Phage family   ICTV / ICTV -0.71764354        2.424846e-03
## 17   host genus  Phage family ICTV / OPTSIL -0.55555556        4.461530e-02
## 18   host genus  Phage family   4K / OPTSIL -0.44785157        1.335140e-03
## 19  host family Phage species   ICTV / ICTV          NA                  NA
## 20  host family Phage species ICTV / OPTSIL          NA                  NA
## 21  host family Phage species   4K / OPTSIL -0.06848238        8.184002e-02
## 22  host family   Phage genus   ICTV / ICTV -0.31833667        5.233900e-04
## 23  host family   Phage genus ICTV / OPTSIL -0.28453759        1.455094e-03
## 24  host family   Phage genus   4K / OPTSIL -0.30883782        1.848653e-07
## 25  host family  Phage family   ICTV / ICTV -0.65218649        6.756223e-03
## 26  host family  Phage family ICTV / OPTSIL -0.53526436        4.639946e-02
## 27  host family  Phage family   4K / OPTSIL -0.50347033        8.258958e-04
```

Examplary command-line calls for BLAST

The command line calls of bidirectional BLAST (blastp or blastn) as done by GBDP prior to the actual calculation of interproteomic GBDP distances are shown below. ### protein-protein BLAST: note: GBDP always compares genomes bidirectionally (two BLAST runs)

```
blastp -task blastp -query 1.faa -db 2.faa -outfmt 5 -num_threads 1  \
       -evalue 1.000000e+01 -word_size 3 \
       -soft_masking true -num_alignments 100000
```

nucleotide-nucleotide BLAST:

note: GBDP always compares genomes bidirectionally (two BLAST runs)

```
blastn -task blastn -query 1.fna -db 3.fna -outfmt 5 -num_threads 1  \
       -strand both -evalue 1.000000e+01 -word_size 11  \
       -soft_masking true -num_alignments 100000
```

Examplary command-line call for FASTME

```
./fastme-2.1.4-linux64 -m b -w b -s -i $EPF_FILE -o $TREE_FILE
```
