# Supplementary figures and images for: VICTOR: genome-based phylogeny and classification of prokaryotic viruses
Source: Bioinformatics. 2017 Jul 7;33(21):3396–404. doi: 10.1093/bioinformatics/btx440 (PMC5860169; doi:10.1093/bioinformatics/btx440)

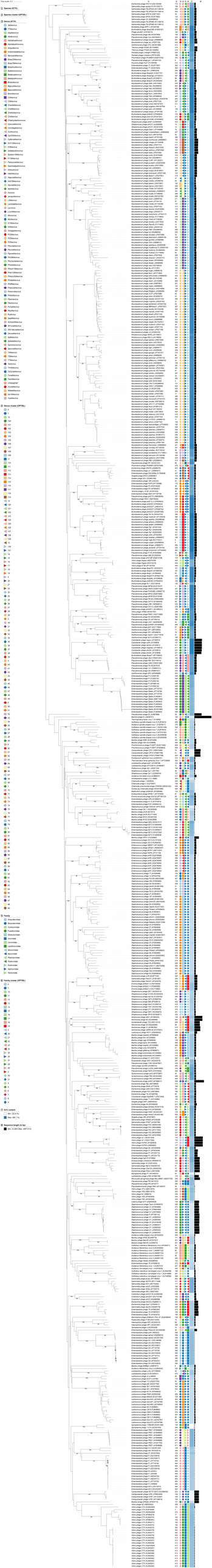

Supplement: Supplementary Data [file btx440_supp.zip › S4_GBDP_tree_nuc_ICTV_dataset_linear.pdf]

 *Ampullaviridae*

 *Corticoviridae*

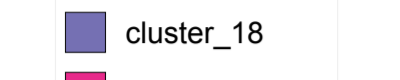

- cluster\_2
- cluster\_20

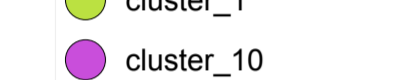

● cluster\_101

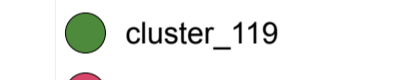

- cluster\_120
- cluster\_121

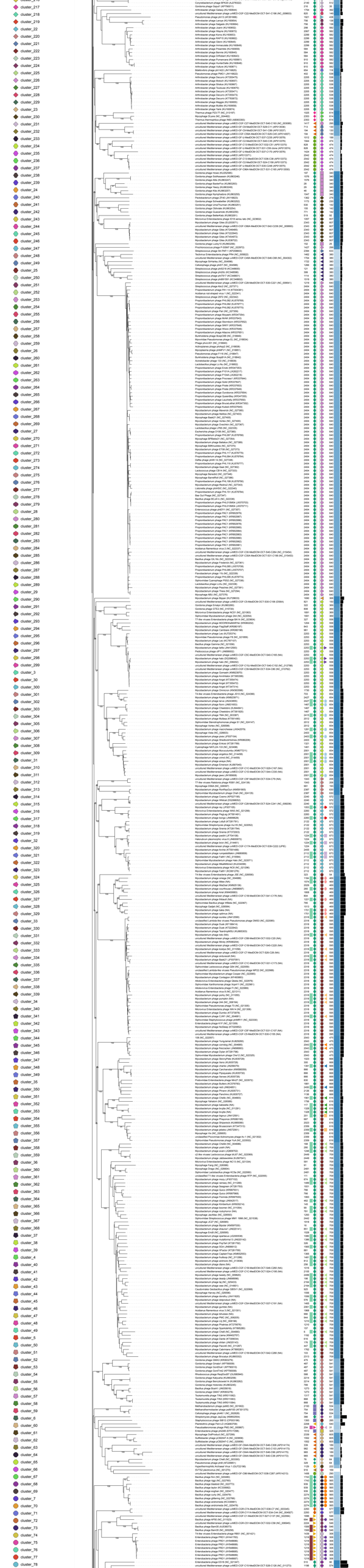

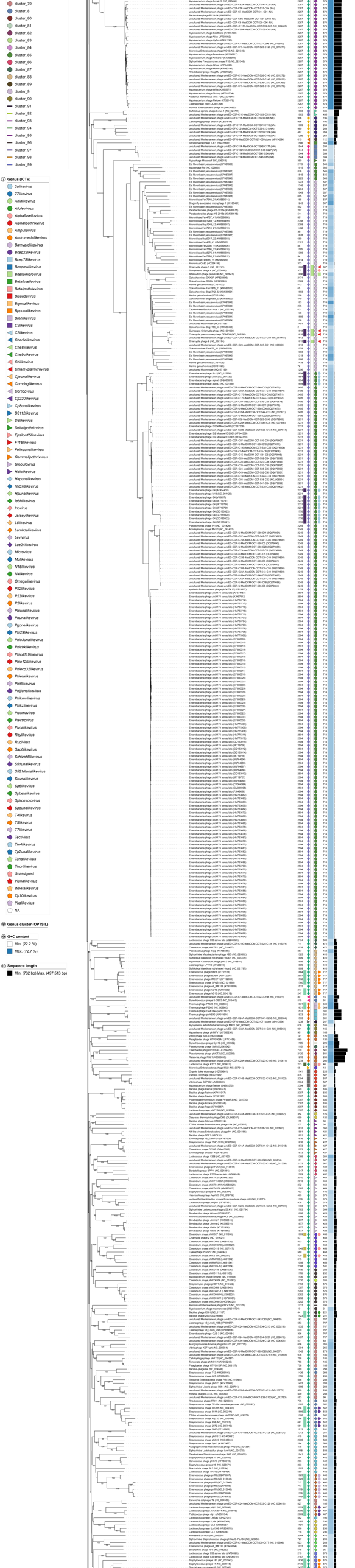

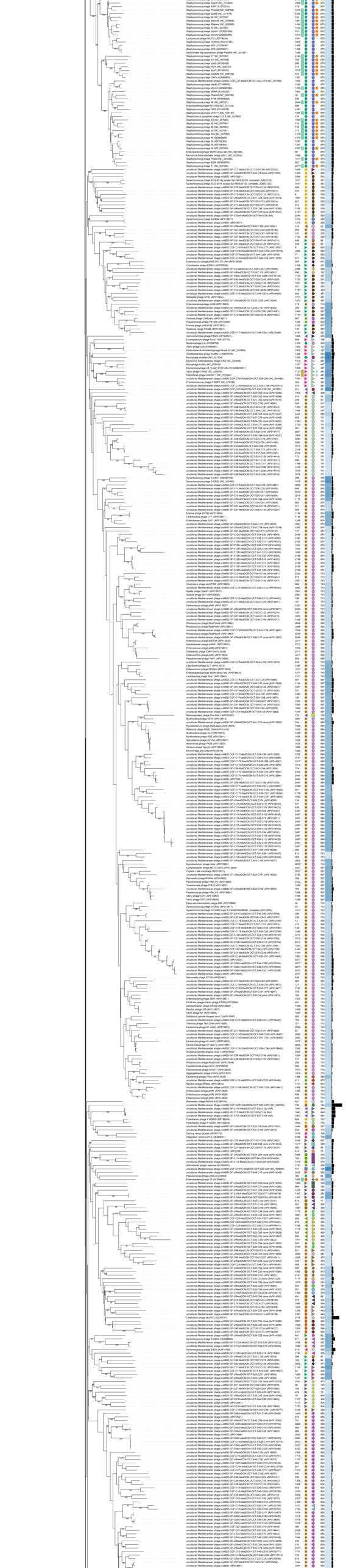

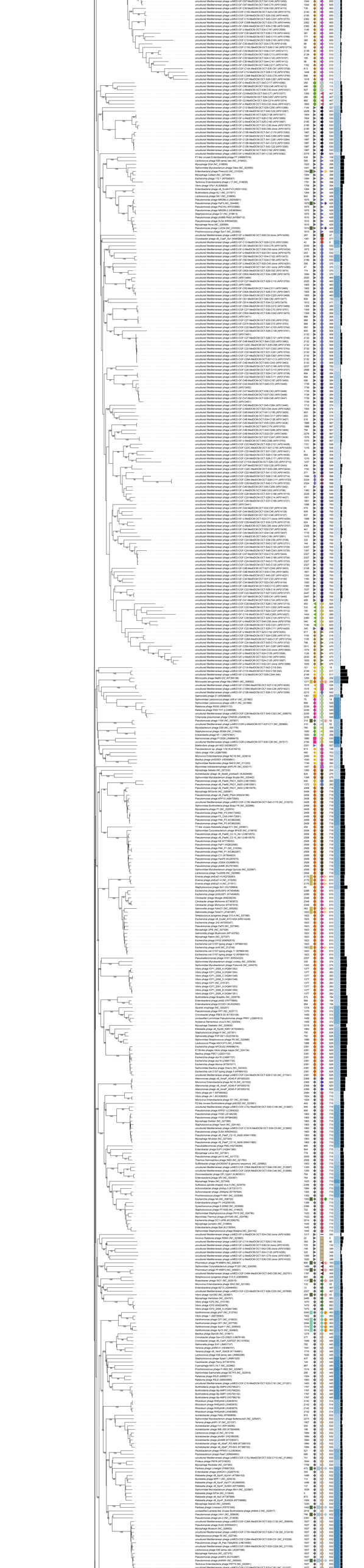

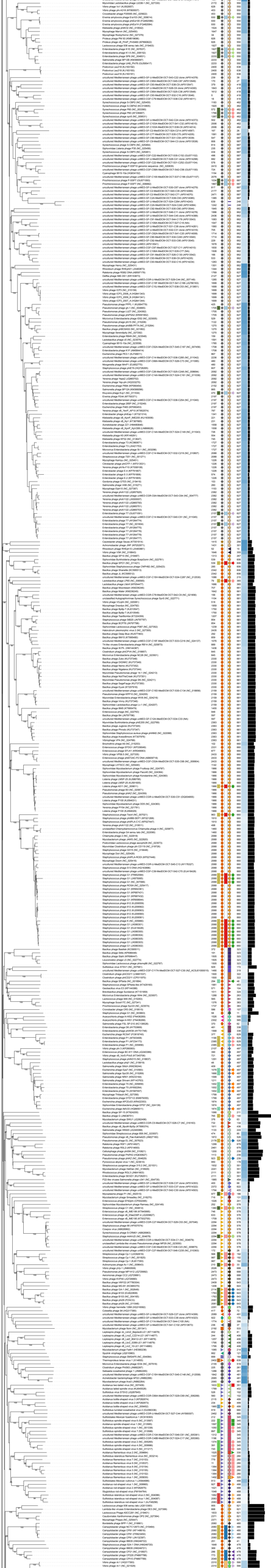

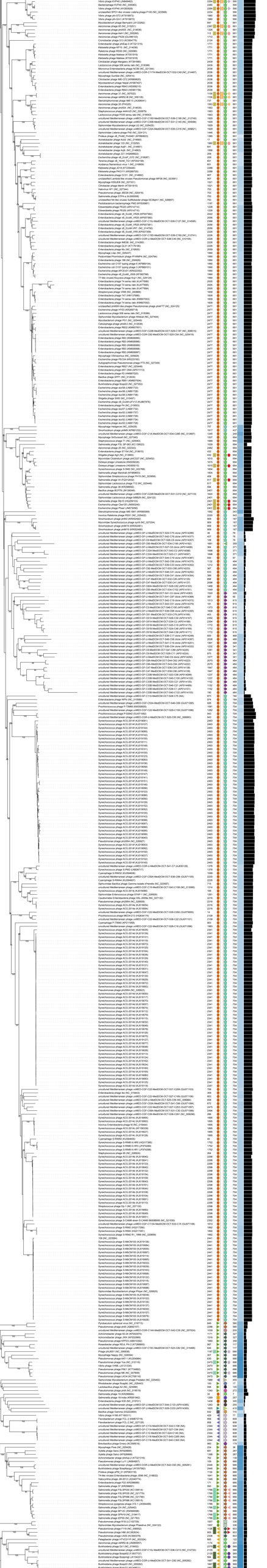

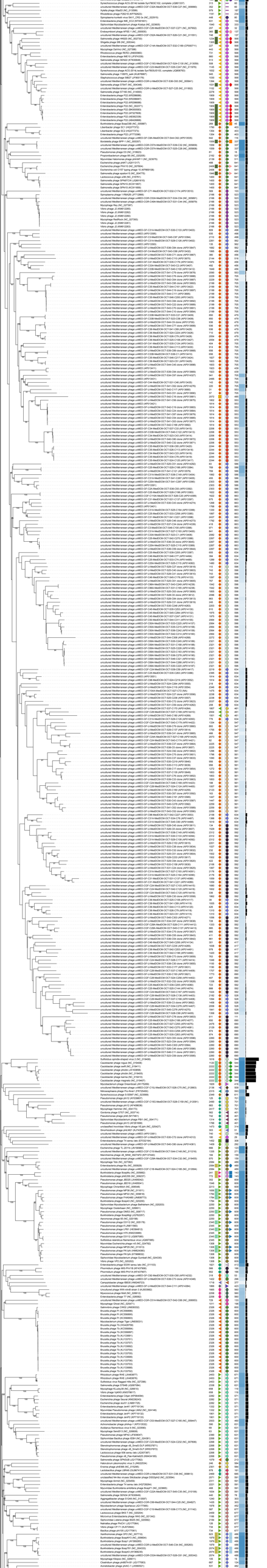

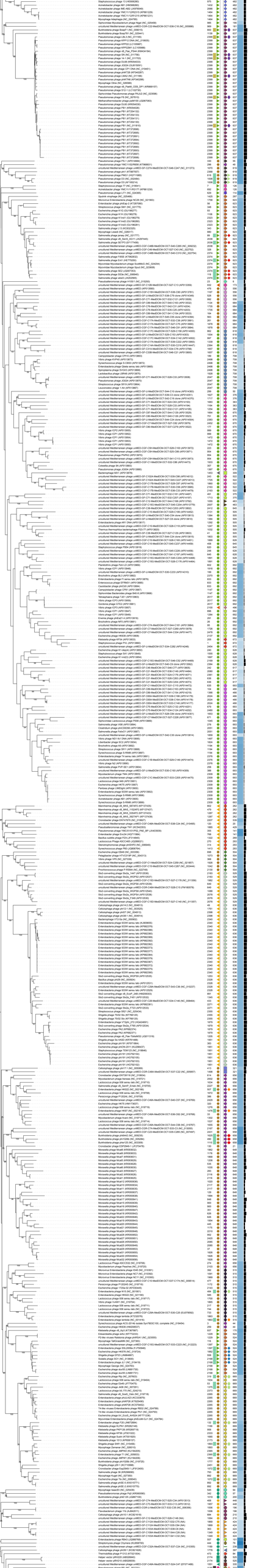

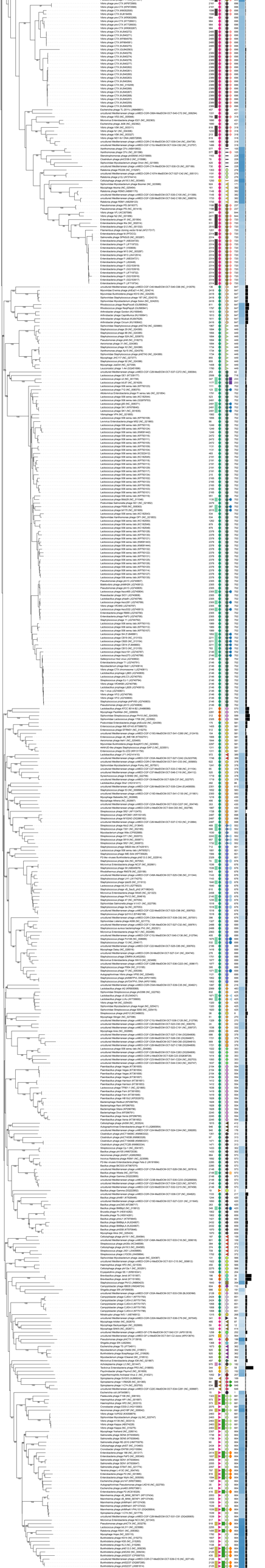

Supplement: Supplementary Data [file btx440_supp.zip › S5_GBDP_tree_prot_extended_dataset_linear.pdf]

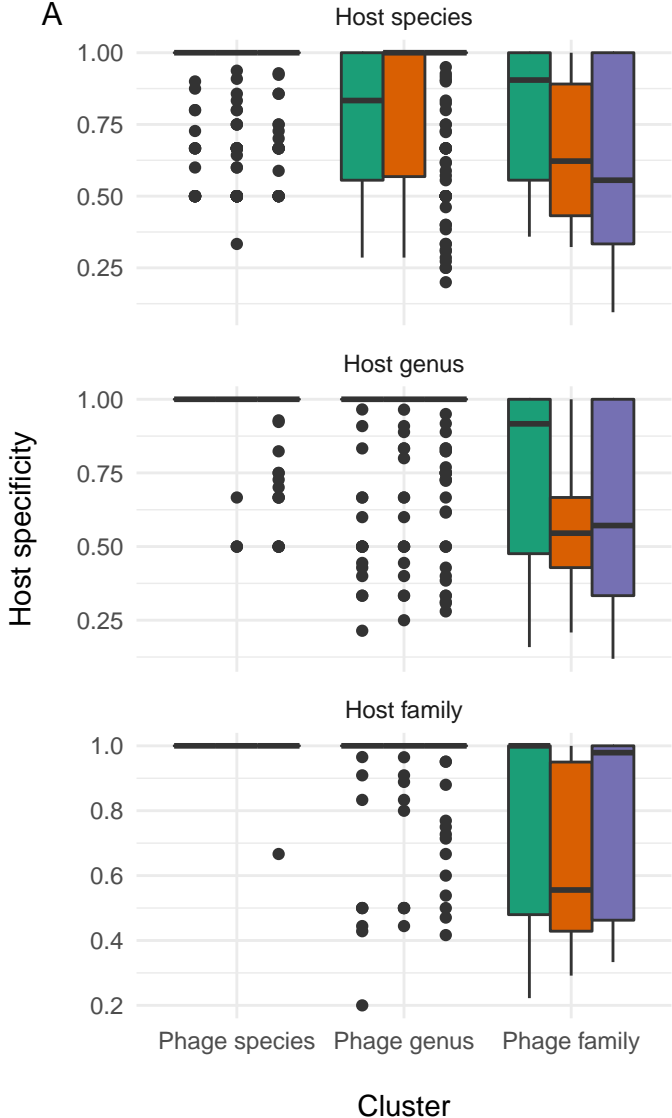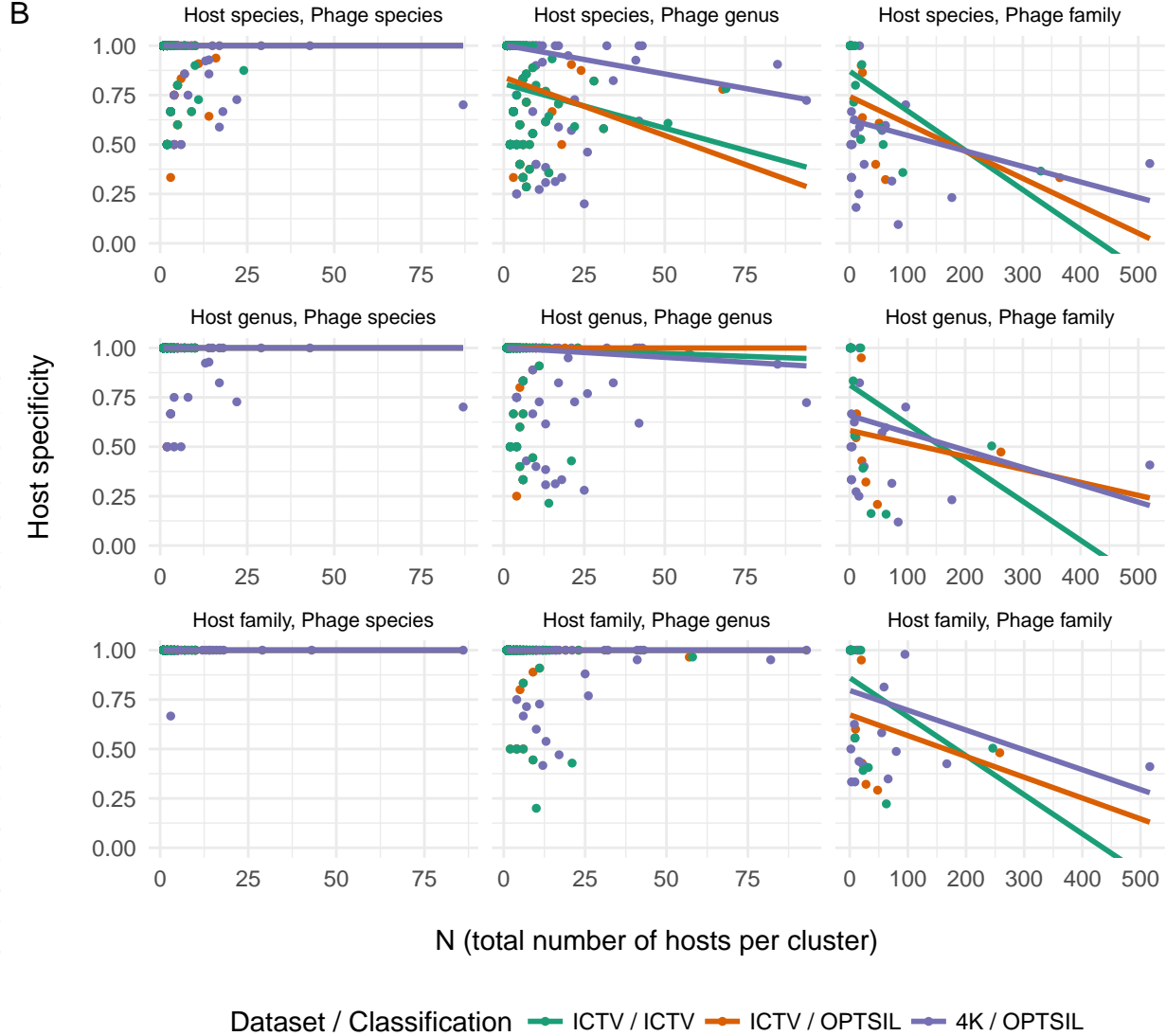

Supplement: Supplementary Data [file btx440_supp.zip › S6_host_specificity.pdf]

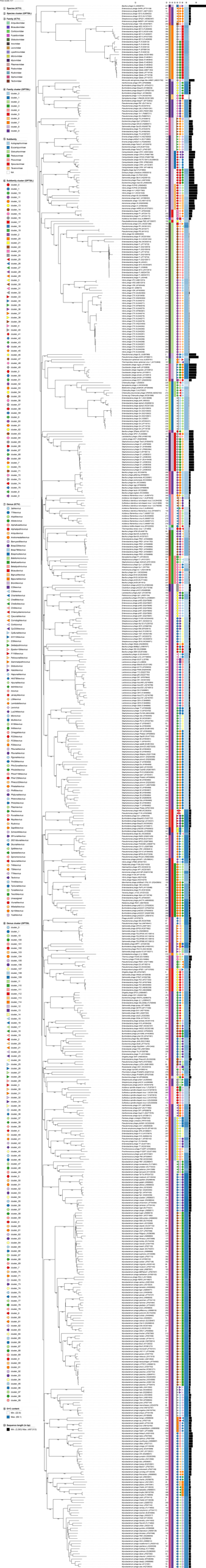

Supplement: Supplementary Data [file btx440_supp.zip › S3_GBDP_tree_prot_ICTV_dataset_linear.pdf]
